# Scoping review of enablers and challenges of implementing pharmacogenomics testing in the primary care settings

**DOI:** 10.1136/bmjopen-2024-087064

**Published:** 2024-11-05

**Authors:** Chun-Wai Mai, Sathvik B Sridhar, Mohammed Salim Karattuthodi, Perishithaa M Ganesan, Javedh Shareef, E Lyn Lee, Keivan Armani

**Affiliations:** 1 Faculty of Pharmaceutical Sciences, UCSI University, Kuala Lumpur, Cheras, Malaysia; 2 RAK Medical & Health Sciences University, Ras Al Khaimah, UAE; 3 Department of Pharmacy Practice, Manipal College of Pharmaceutical Sciences, Manipal Academy of Higher Education, Manipal, Udupi, Karnataka, India; 4 Department of Pharmacy Practice, IMU University, Kuala Lumpur, Malaysia; 5 IMU University, Kuala Lumpur, Malaysia; 6 Department of Primary Care and Public Health, School of Public Health, Imperial College London Faculty of Medicine, London, UK; 7 UCSI University Faculty of Pharmaceutical Sciences, Cheras, Kuala Lumpur, Malaysia

**Keywords:** Primary Health Care, Health Services, Health Workforce, Review, Therapeutics

## Abstract

**Abstract:**

**Introduction:**

Pharmacogenomic testing (PGx) plays a crucial role in improving patient medication safety, yet ethical concerns and limitations impede its clinical implementation in the primary care settings.

**Aims:**

To systematically review the current state of PGx in the primary care settings and determine the enablers and challenges of its implementation.

**Design:**

A scoping review was carried out by adhering to Arksey and O’Malley’s 6-stage methodological framework and the 2020 Joanna Briggs Institute and Levac *et al.*

**Data sources:**

Cochrane Library, EMBASE, Global Health, MEDLINE and PubMed were searched up to 17 July 2023.

**Eligibility criteria:**

All peer-reviewed studies in English, reporting the enablers and the challenges of implementing PGx in the primary care settings were included.

**Date extraction and synthesis:**

Two independent reviewers extracted the data. Information was synthesised based on the reported enablers and the challenges of implementing PGx testing in the primary care settings. Information was then presented to stakeholders for their inputs.

**Results:**

78 studies discussing the implementation of PGx testing are included, of which 57% were published between 2019 and 2023. 68% of the studies discussed PGx testing in the primary care setting as a disease-specific themes. Healthcare professionals were the major stakeholders, with primary care physicians (55%) being the most represented. Enablers encompassed various advantages such as diagnostic and therapeutic benefits, cost reduction and the empowerment of healthcare professionals. Challenges included the absence of sufficient scientific evidence, insufficient training for healthcare professionals, ethical and legal aspects of PGx data, low patient awareness and acceptance and the high costs linked to PGx testing.

**Conclusion:**

PGx testing integration in primary care requires increased consumer awareness, comprehensive healthcare provider training on legal and ethical aspects and global feasibility studies to better understand its implementation challenges. Managing high costs entails streamlining processes, advocating for reimbursement policies and investing in research on innovation and affordability research to improve life expectancy.

**STRENGTHS AND LIMITATIONS OF THIS STUDY**

- The consultation sessions with the stakeholders were conducted to co-develop the research questions, to sense-check the findings and to consolidate the discussion points pertinent to the findings.
- Grey literature that was not peer-reviewed, was not included in the study.
- A plausible limitation was the lack of critical appraisal of the included studies for their quality in this review, despite the fact that critical appraisal is not required for scoping reviews.

## Background

 Pharmacogenomics (PGx) broadly defines how genomic variation affects a patient’s response to a drug.^1^ Distinct polymorphisms in drug-metabolising enzymes and drug transporters were a foundation for PGx.^2^ With the advance in health technology, the 2000 collaborative effort to draft the human genome marked a turning point, followed by the International Single Nucleotide Polymorphisms Map Working Group’s efforts to map variations in the human genome sequence.^2 3^ More importantly, the advancement of health technology has positioned PGx as a key component in the field of personalised medicine. The application of health technology has ranged from rationalising mutation-specific therapies to personalising early detection strategies, disease prevention and treatments, have been increasingly used in both clinical settings and research contexts based on individual patient profiles.^4^ This approach tailors medical treatment to an individual’s unique genomic makeup to improve treatment outcomes and minimise adverse effects.^5^ While PGx testing provides useful information by detecting genetic variants that impact medication metabolism and response, it is not ideal for all patients.^6^ PGx testing can help guide the selection of drugs that are more likely to be beneficial and have fewer adverse effects depending on an individual’s genetic makeup.^7^ However, it does not consider other important aspects, such as the influence of environment, comorbid diseases and patient adherence, which can substantially impact treatment results. As a result, while PGx testing is an effective tool for customising therapy, it should be used with extensive clinical judgement rather than as a sole predictor of optimal treatment.^8^ This approach tailors medical treatment to an individual’s unique genomic makeup to improve treatment outcomes and minimise adverse effects.^5^

Individual genetic variations play a significant role in influencing the effectiveness and safety of medications. Genetic differences in drug-metabolising enzymes, transporters, receptors and other therapeutic targets have been related to interindividual variances in the efficacy and safety of several frequently prescribed medications such as antidepressants (eg, selective serotonin reuptake inhibitors and anticoagulants (eg, warfarin), which account for approximately 20–30% of medication response variability.^9^ Genetic differences do not follow a consistent pattern among populations. Instead, they show significant variation within and between different geographical ancestries.^10^ For example, specific PGx variants that impact drug metabolism are more commonly found in certain populations, leading to variations in drug response and the occurrence of adverse effects. Acknowledging and understanding these genetic variations specific to different populations is essential for the successful application of personalised medicine. This knowledge enables clinicians to customise treatments that are safe and effective for a wide range of patients.^11 12^ Inter-individual genetic differences within and between geographical ancestry contribute significantly to medication response variability and are linked to variants affecting the pharmacokinetics and pharmacodynamics of drugs.^13 14^ The British Pharmacological Society and the Royal College of Physicians have urged patients to be examined for genetic variations that can impact their respond to commonly used drugs.^15^ The US Food and Drug Administration (FDA) recommends genetic screening before using certain medications.^16^

Developing countries are the strongest users of PGx-guided therapy.^17^,^20^ However, the utilisation of PGx across Europe varies.^21^,^23^ The public seemed to prefer and opt for PGx testing, especially those with chronic diseases.^24^ Gene-drug interaction variability within the European population has been established and has thus increased the scope for PGx.^25^

An observational study from the UK discussed the implementation of PGx testing in secondary care for high-risk medications. The authors emphasised the need for broader application in primary care owing to the high prescribing tendency in the community.^26^ The adoption of PGx testing services in different healthcare settings has varied owing to a multitude of factors, including the promotion of appropriate and evidence-based medication usage, ethical considerations, legal implications, healthcare provider and patient education, support for electronic health records, clinical utility and validity of test outcomes, accessibility, regulatory frameworks, as well as availability and affordability.^2027^,^30^ The cost implications of PGx testing depend on the insurance coverage offered by companies. Few insurance firms offer coverage for PGx testing, and those that do must follow strict guidance and policies to justify and approve requested PGx tests.^31^ This can affect the preference for pre-emptive PGx and reactive PGx testing.^32^ Both pre-emptive and reactive testing have been found to be cost-effective in different disease states or clinical care contexts and positively impact patient outcomes.^33^

The US FDA has emphasised the importance of PGx testing for drug discovery, development and treatment of patients. 500 different biomarkers concerning drugs have been stated in their public domain.^34^ Similarly, the European Medicines Agency has guidelines regarding the use of PGx testing during drug approval processes.^35^ Despite the regulatory authorities’ new recommendation to incorporate PGx testing in the drug approval process, testing regarding marketed products is also not a routine practice. Moreover, patients were also disrupted from subscribing to the PGx testing due to the availability of resources and many hindrances factors that may vary across the nation.^36^

While PGx testing offers several benefits, it is important to acknowledge the ethical concerns surrounding it, especially in a primary care setting. Ethical dilemmas may emerge due to the potential misuse of informed consent in genomic testing, including the potential dangers, risk, harms and consequences associated with genomic information.^37 38^ Additionally, genomic information may raise questions about ownership, access rights, affordability, fiduciary responsibility, respect and the possibility of discrimination.^37 38^ Furthermore, there are concerns about the administering PGx testing among vulnerable communities. Assessing the potential long-term implication of identifying genomic variability in different categories of vulnerable population may raise ethical concerns.^37^,^39^

References in the literature provide evidence for PGx testing in primary care. Through prospective trials, it has been demonstrated that when paired with comprehensive medication management services and point-of-care clinical decision support systems, improvised drug prescribing lessened the burden of mental illness, thereby enhancing clinical outcomes.^40^ Barriers such as a perceived lack of knowledge on acceptance, scalability and implementation and insufficient evidence of therapeutic outcomes improvement have been reported.^41^ Financial constraints and the knowledge and abilities of healthcare professionals hinder implementation.^42^

Moreover, since the interpretation of genomic information is still evolving, inadequate inferences or confounding factors may cause healthcare providers to opt for incorrect treatment, complicating the ethical landscape and raising public concern about their health.^43^ While PGx testing offers positive benefits, it is important to acknowledge the concerns related to this practice, especially in a primary care setting. Thus, this scoping review was conducted to systematically review the current state of PGx in the primary care and determine the enablers as well as challenges of implementing PGx testing in primary care settings.

## Methods

A scoping review was carried out by adhering to Arksey and O’Malley’s 6-stage (step 1 to step 6) methodological framework and the 2020 Joanna Briggs Institute.^44 45^ Covidence, a web-based collaboration software platform designed to facilitate carrying out reviews such as systematic reviews and scoping reviews, was used for the review.^46^ Further, Levac and colleagues’ recommendations were applied to maximise the methodological rigour and, thus, reported the details of the six stages under the following subheading.^47^ The Preferred Reporting Items for Systematic Review and Meta-Analyses extension for Scoping Reviews checklist was used to guide the reporting of this review.^48^

### Identifying the review question

CWM, an expert in the field of PGx, and KA, a primary care research expert had the initial discussion about the potential review questions that could address some of the gaps in the current literature on PGx testing and its applications in primary care settings. All authors are academics who joined the subsequent discussions, clarified the aims and objectives of the scoping review and collectively agreed on the following review question: ‘What are the enablers and the challenges of implementing PGx testing in primary care settings?’

### Identifying the relevant studies

The authors agreed on the search strategy with no limits on publication dates. The search was concluded on 17 July 2023 based on the predetermined search strategy (online supplemental file 1). We consolidated the search resources following advice from a subject librarian to ensure a wide range of relevant databases such as Cochrane Library, EMBASE, Global Health, MEDLINE and PubMed. The International Prospective Registry of Systematic Reviews (PROSPERO) was also reviewed for any similar studies, both ongoing or completed, to avoid any potential duplication. Articles in English were only considered due to a lack of resources for translating studies. The inclusion and exclusion criteria were finalised through an iterative process to allow necessary refinements following initial searches (see table 1).

**Table 1:** Inclusion and exclusion criteria

Criterion	Inclusion	Exclusion
Period	Any	-
Literature	Peer-reviewed articles	Review articles of any type, non-peer-reviewed academic articles.
Geographical location	Any	–
Setting	Primary care settings	Secondary and tertiary care settings.
Study focus	Information on the pharmacogenomics testing implementation in primary care settings	No information is directly related to the implementation of pharmacogenomics testing in primary care settings.
Study design	All types of qualitative and quantitative studies, clinical audits	All types of reviews, including systematic reviews, meta-analysis.
Language	English	Other languages than English.

### Selecting the studies

Articles were identified across five databases, which were exported into Covidence for further processing. Two reviewers independently screened each article, and a third reviewer resolved any discrepancies.

### Charting the data

Data charting facilitates the transfer of the relevant information from the selected articles into a data extraction table.^5^ The authors created a data extraction template using the Covidence extraction template. The data extraction template was contextualised to meet the study objectives and the research questions proposed at the beginning of the review, which contained standard information such as title, lead author, type of study, aims, objectives, key stakeholders, findings in relation to the enablers and the challenges of implementing PGx in the primary care settings and recommendations. All authors were involved in charting the data, and PMG carried out most data extraction. Although data extraction needed one reviewer per article, KA checked each article’s extraction data for final approval.

### Collating, summarising and reporting the results

KA and PMG synthesised the results by collating and summarising the findings following data charting. Results were then presented to the rest of the authors for their comments and interpretations. The authors were registered pharmacists who had the experience of practicing in primary care settings. They discussed the results from the practice and policy’s point of view. The authors did not carry out a quality assessment exercise as scoping reviews do not normally need an appraisal for quality and bias due to their descriptive nature.^6^

### Consulting stakeholders

Although stakeholders’ involvement and consultation are not mandatory stages for conducting scoping reviews, we involved a subset of stakeholders who were available to us in two stages. These stakeholders were the primary care physicians (PCPs) or community pharmacists who were elected leaders in their respective professional societies and had at least 10 years of primary care clinical experience. Invitations were sent by the research team to all eligible stakeholders. All stakeholders who declared no conflict of interests with any PGx service provider were to participate. We conducted a brainstorming session with these stakeholders. The 10 stakeholders were from independent or chain medical clinics (n=5) or community pharmacies (n=5). We then presented the findings to them for their comments and feedback.

### Patient and public involvement

There was no patient or public involvement in addition to the above-mentioned stakeholders.

## Results

**Figure 1:**
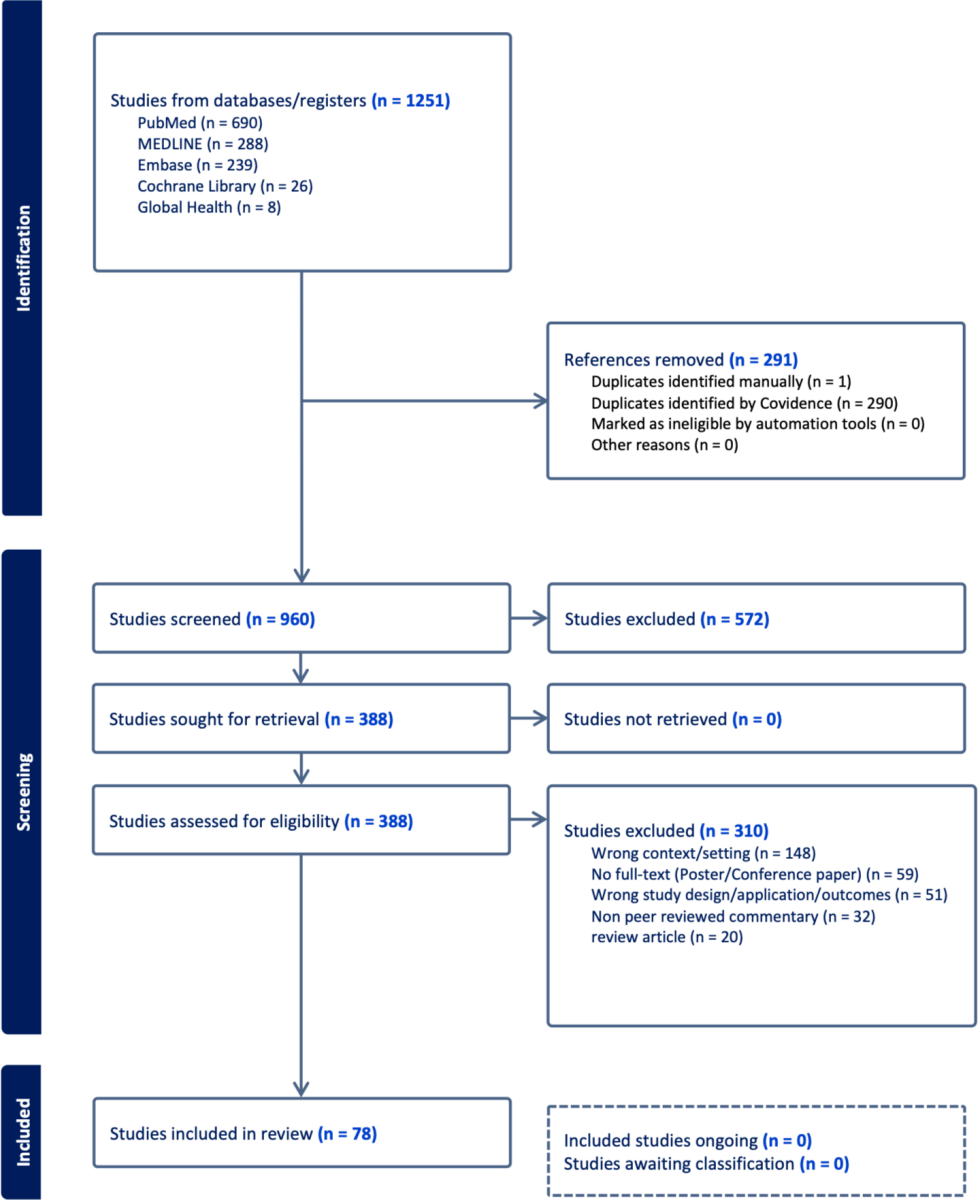
Flow diagram of the scoping review.

A total of 1251 articles were initially identified across five databases, that is, PubMed (n=690), MEDLINE (n=288), EMBASE (n=239), Cochrane Library (n=26) and Global Health (n=8). 291 duplicates were removed, leaving 960 articles for title and abstract screening. A total of 378 articles met the inclusion and exclusion criteria for full-text screening. We present the findings from 78 studies on different aspects of PGx testing implementation in primary care settings, such as stakeholders’ views and involvement, enablers and challenges of implementing PGx testing (online supplemental file 2). The PGx testing in the primary care setting in these studies was discussed either as disease-specific themes (n=53), such as mental health conditions, cardiovascular conditions, diabetes or population-specific themes (n=11), such as general patient population, paediatric and geriatric patient population or public health themes (n=3) and others not specified (n=11). The full-text screening eliminated 290 articles because of wrong context/setting (n=148), no full-text availability, for example, for poster/conference papers (n=59), wrong study design or application or outcomes (n=51) and non-peer-reviewed commentary (n=32) and thus, 78 studies were included in the final review on which results are reported (figure 1).

### Publication date

The earliest publication was in the year 2007, and the latest publication was in 2023 when data collection ended. More than half of the studies (57%) were published in the period between 2019 to date. Nearly one-third (n=22) of studies were published between the years 2016 and 2018. The number of publications has increased significantly in the last 6 years, that is, between 2018 and 2023.

### Types of studies and location

**Figure 2:**
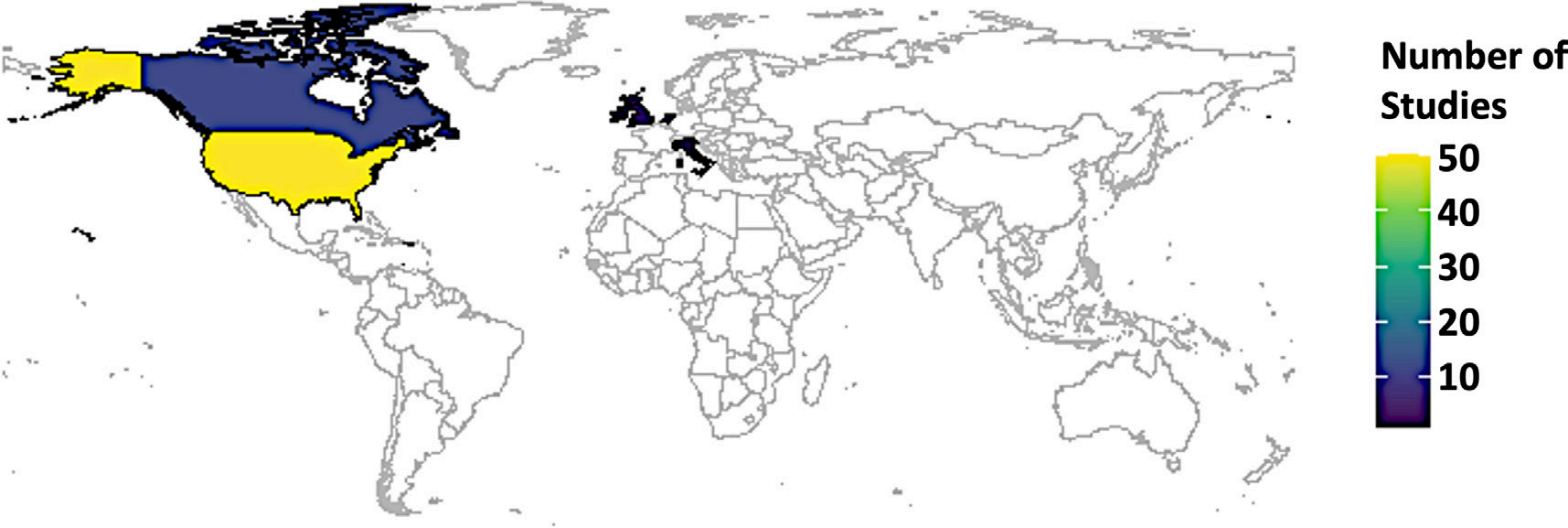
Country of origin of the articles included in this review.

A wide array of study designs was pulled together in this review, ranging from commentaries (n=2) to qualitative studies (n=7) to quantitative studies (n=16), including randomised controlled trials (n=5) to mixed methods studies (n=54). An overwhelming majority of the studies were from the global north (n=77), for example, 51 studies from the USA and its territory, 12 studies from Canada, 14 studies from the European Union, while there was only one study from Singapore (figure 2). The study types can be categorised into quantitative studies (n=16) and mixed method studies (n=54). Quantitative studies can be further divided into (1) randomised controlled trials (n=5), where the controlled experimental settings were used to assess the efficacy of PGx testing; (2) cohort studies (n=4), where these groups were monitored over time to evaluate the outcomes of PGx testing; (3) cross-sectional surveys (n=3), where one-time data collection methods were used to evaluate respondents’ beliefs, expertise and PGx-related behaviour; (4) case–control studies (n=2) where the effects of PGx testing were examined by comparing individuals with particular results to those without; and (5) pre–post intervention studies (n=2), where the outcomes were examined both before and after PGx testing was used.

In addition, mixed methods studies (n=54) can be further categorised into (1) explanatory sequential designs (n=15), where quantitative data were gathered first, followed by qualitative data to explain the quantitative results; (2) exploratory sequential designs (n=20), where quantitative data were collected after conducting qualitative research to create or refine hypotheses; and (3) convergent parallel designs (n=19), where qualitative and quantitative data were gathered concurrently, the finding were compared and comprehensive conclusions were drawn. This thorough analysis addresses the variability within the broader categories of quantitative and mixed methods research, providing a deeper understanding of the studies covered in the study.

### Stakeholders

From the selected literature, the stakeholders included the service users/patients, members of the public, healthcare professionals including general practitioners, physicians, pharmacists, nurses, physician assistants, public health consultants/professionals, geneticists, phlebotomists, genetic counsellors, mental health providers, obstetricians, gynaecologist, psychiatrists and cardiologist. Most of the stakeholders were PCPs (n=43), followed by pharmacists (n=32), allied healthcare professionals (n=27) and primary care providers who were not specified (n=15) (online supplemental file 3). Additionally, there was general agreement with the results when they were presented to a panel of stakeholders (n=10) whom we had individually consulted for this study.

### Current status of pharmacogenomics testing in the primary care settings

To understand the current status of PGx testing in primary care, we classified the key conclusion of these studies into three categories, namely the (1) favourable view in which the key conclusion supports PGx implementation in primary care; (2) not favourable, in which the key conclusion does not support PGx implementation in primary care; and (3) neutral views in which the study did not provide a clear stance on supporting or not supporting PGx implementation in primary care. More than half (52%) of the studies had favourable views toward the status of PGx testing in primary care settings, whereas 43% of the studies had unfavourable views and 5% of the studies offered neither favourable nor unfavourable views (online supplemental file 4). Most of the favourable views stemmed from the perceived benefits of PGx testing to the patient’s clinical outcomes, selection of the most precise treatment modality, decrease in the incidences of adverse drug reactions due to polypharmacy and improved medication adherence.^49 50^ Other favourable opinions were the health systems level benefits of PGx testing, such as lowering the healthcare costs and broader applicability of PGx in the areas of preventive care, population health and community health interventions.^51^

The main reasons for unfavourable opinions were the perceived lack of information or findings on the acceptability, scalability and implementation aspects of PGx testing in primary care settings. Furthermore, the perceived limited evidence of the effectiveness of PGx testing on impacting clinical outcomes, limited knowledge and skills of the healthcare professionals to operationalise PGx testing in the routine delivery of care as well and financial concerns, data security were some of the unfavourable concerns to implementing PGx testing in the primary care settings.^49 52 53^ Specifically, Türkmen *et al* highlighted PGx results could be guided by databases such as PharmGKB, which contains studies with low or moderate level of evidence. They also noted that the study design, with qualitative studies not being meant for generalisability of the findings, along with factors such as diverse ethnicity, heterogeneity, poor compliance to medication, statistical bias and publication bias, may further limit the implementation of PGx in primary care.^49^

### Enablers of PGx testing implementation in the primary care setting

The benefits of using PGx testing in primary care settings were discussed in almost all studies (n=77). PGx testing implementation was facilitated by three main factors, broadly: (1) diagnostic and therapeutic benefits in collaborative practice; (2) reduction in healthcare costs; and (3) empowering healthcare professionals to deliver their clinical services, especially for the physicians and community pharmacists. A total of 23 studies reported other possible enablers, including programmes that support clinical decision-making, precision medicine, personalised medicine, individualised care, drug–drug interactions, patient safety and optimal medication use.

#### Diagnostic and therapeutic benefits in collaborative practice

Around 10% (n=12) of the studies reported the findings that PGx supports collaborative clinical practice by allowing a precise choice of therapeutic agents in treating patients. For example, findings from a primary care precision medicine clinical offering PGx services at the University of Pittsburgh Medical Centre Health System showed that genotype-guided clinical decisions successfully supported the primary care providers’ adoption of genetic information to guide statin therapy in routine clinical practice.^54^ A UK study described the benefits of PGx testing to support personalised medicine and the management of calcium channel blocker side effects through genomic-guided information on pharmacogenetic variations.^55^

#### Reduction in healthcare costs

The potential for cost-saving associated with the implementation of PGx testing was mentioned in 20% of the studies (n=15). Various cost-saving approaches were proposed, namely (1) economic evaluations; (2) stakeholders perceptions; and (3) indirect evidence. Formal economic evaluations were used in several studies to determine whether PGx testing was cost-effective. Cost-utility, cost-benefit and cost-effectiveness studies were frequently performed as part of these assessments. For example, when PGx testing guided drug selection and dose decisions, a randomised controlled trial found lower healthcare expenditures due to fewer adverse drug events (ADEs) occurred. This study measured the financial gains connected with fewer ADEs and hospitalisations using a cost-effectiveness methodology.^56^ An economic evaluation approach was employed in prospective cohort research conducted in Singapore to evaluate the effects of a PGx-based medical decision support system on healthcare expenditures and quality. The study showed that by enhancing medication dosage and improving treatment results, PGx testing led to cost-savings.^57^

In term of stakeholder perceptions, some research examined cost-savings from the viewpoint of stakeholders, such as legislators and healthcare professionals, in addition to economic evaluations. Stakeholders believed that PGx testing could be an effective way to reduce overall healthcare costs by minimising trial-and-error prescribing and the adverse drug experiences that come with it. Qualitative interviews with PCPs, for instance, revealed that PGx testing could save long-term expenses by enabling more accurate medication administration. Alternative approaches would be through indirect evidence. A few studies highlighted improvements in patient outcomes that were associated with lower healthcare utilisation, which served as an indirect source of cost-saving data. These studies suggested that more targeted treatments resulting from PGx testing could reduce total healthcare costs by avoiding the need for extra interventions, even though they did not conduct direct economic evaluations.

#### Empowering healthcare professionals to deliver their clinical services

Over 28% of studies emphasised the importance of incorporating healthcare professionals such as community pharmacists, to improve patient care through implementing PGx in a primary care setting. The advantages of involving community pharmacists in administering PGx testing include (1) enhanced medical management, (2) increased accessibility and patient engagement, (3) better integration with clinical decision support systems and (4) increased physician adoption of PGx. By using PGx testing, community pharmacists can customise more drug regimens based on each patient’s unique genetic profile, leading to fewer adverse drug reactions and increased efficacy. An open-label, non-randomised observational trial reported better patient outcomes from community pharmacists based PGx screening, since pharmacists could efficiently provide more input on the regimens.^58^

In addition, patients’ accessibility to PGx testing is increased when it is incorporated into community pharmacy practices, especially in underprivileged areas. Research indicated that patients who experienced easier access to genomic services via their neighbourhood pharmacies, were likely to have thoughtful and educated conversations regarding their treatment options.^59^ Community pharmacists play a crucial role in helping patients understand the meaning of PGx test results. Patients would then adhere to the individualised treatment programmes when they are more educated about how genetic information can guide their pharmaceutical choices.

Interestingly, including PGx testing in a clinical decision support system (CDSS), greatly enhanced its efficacy. Research indicated that community pharmacists who used CDSSs in combination with PGx testing were more capable of making well-informed choices regarding medication dosage and therapy modifications. This integration reduces the possibility of drug errors and helps provide more accurate recommendations.^59^ Moreover, physician preference for PGx in patient care has increased due to the convenience of receiving PGx services through community pharmacists. By collaborating with pharmacists, who perform PGx testing, physician can focus on better decision-making and ultimately improves patient outcomes.^60^

### Challenges of pharmacogenomics testing implementation in the primary care setting

The challenges of implementing PGx testing in primary care settings were discussed in all studies (n=78). There were four main areas of challenge: (1) dearth of data on the scientific evidence such as clinical-genomic databases; (2) lack of bespoke PGx training modules/courses for the healthcare professionals to apply the PGx testing principles; (3) dearth of data on patient awareness and acceptability of the use of PGx testing in patient care; and (4) high costs associated with PGx testing.

#### The dearth of data on scientific evidence, such as clinical-genomic databases

45% of the studies (n=35) reported the lack of solid scientific evidence to produce reliable clinical-genomic databases and clinical practice guidelines (n=35), followed by perceived publication bias (n=23) in the studies in the field of PGx. For example, a 2017 study highlighted that a constraint of the study was the limited sample size, which might have introduced bias as the findings might not accurately reflect the viewpoints of all PCPs or those within the chosen primary practice sites.^61^ Almost a quarter (n=18) of the studies also acknowledged that their studies may had the inevitable recruitment bias, which could limit the potential to immediately implement PGx findings across all populations in primary care settings.

#### Lack of bespoke PGx training modules/courses for the healthcare professionals

The insufficiency of appropriate training for primary care providers to administer PGx testing was a notable obstacle identified in 17 studies. Each healthcare practitioner has distinct PGx training. Due to their limited exposure to genetic concepts and how they are applied in daily practice, many PCPs report feeling unprepared to use PGx testing. PCPs need comprehensive primary care training to evaluate PGx test results and incorporate them into clinical decision-making. Training courses must concentrate on managing drug-gene interactions, using genetic information to inform medication selection and dosage and clearly communicating findings to patients. Nurse practitioners’ capacity to offer effective patient education and individualised medication management is hampered by their lack of PGx testing-specific training such as data analysis, and the incorporation of PGx data into patient care plans. In addition, the limited availability of specialised training programmes for pharmacists also hinders their ability to apply PGx testing in their practice.^62^ Specific trainings for pharmacists should include interpreting of genetic data, applying PGx in drug therapy management and integrating into pharmacy practice. The inadequacy of customised training programmes for these diverse healthcare worker groups limits their ability to apply PGx testing in primary care environments. Addressing this gap with focused educational initiatives is essential to optimising the benefits of PGx technology.

#### The dearth of data on patient awareness and acceptability of the use of PGx testing

Around 10% of the studies reported the dearth of data on patient awareness and patient acceptability of the PGx testing as a barrier to the implementation of PGx testing in primary care settings. For instance, a 2017 study showed the importance of patients’ willingness to consent to be involved in clinical-genomic treatment modalities, which would need patients to be fully aware of the technical aspects of PGx testing, including ethical aspects.^63^ A qualitative study revealed that patient anxiety and fear of disclosing genetic information to a third party was the main barrier to the implementation of PGx testing in primary care settings.^62^

#### High costs associated with PGx testing

Almost 20% (n=14) of the studies mentioned high costs associated with PGx testing in primary care settings. Insurance coverage, out-of-pocket expenditure and institutional return of investment—investment in setting up PGx testing—were among the points raised in regards to the costs and who should bear the cost based on the healthcare systems in the global north, Western Europe and Australasia.^5 64 65^

## Discussion

PCPs play a key role in incorporating PGx into standard clinical practice. Primary healthcare professionals need to educate patients on the importance of genetic data and how it affects individualised treatment plans. Collaboration with genetic counsellors and other medical professionals can also help maximise the use of PGx in patient care. Genetic counsellors assist individuals and healthcare providers in better understanding intricate genetic details. ^63^

Collaboration among academia, healthcare, industry and regulatory agencies is essential for integrating PGx into clinical practice.^66 67^ PGx has been effectively integrated into healthcare systems in both the USA and the UK. There is significant variation in the implementation of PGx across Europe^21^ and Gulf Cooperation Council countries like Saudi Arabia, UAE and Qatar.^17 18^ PGx has made significant progress in the UK, with the National Health Service supporting genetic screening to enhance medication therapy.^22^ Similarly, it is also used in Australia and Canada to enhance the optimal clinical decision.^68 69^ On the other hand, there is a rise in the PGx utility in Singapore, Japan, South Korea and China, particularly for chronic diseases.^19 57 70^ Some regions still face complex regulatory structures and ethical issues, and this is a big challenge.^71^ Regulatory agencies’ well-defined guidelines give healthcare providers confidence and create an environment in which PGx practices are not only acceptable but actively promoted.^72^ The regulatory environment is greatly influenced by policymakers, who make sure that it permits a smooth integration of PGx into standard primary care practice and keeps pace with the field’s rapid evolution.

Several studies emphasise the importance of PGx testing in cardiovascular diseases and neuropsychiatry disorders^2356 73^,^75^ due to its ability to choose more precise treatment modalities, a reduction in adverse drug reactions caused by polypharmacy and a significantly improved medication adherence.^74 76 77^ However, the dearth of data on scientific evidence, particularly in areas such as clinical genomic databases, poses a significant challenge for PGx testing. One of the obstacles is the limited availability of high-quality genomic data linked to clinical outcomes.^78^ Clinical genomic databases that integrate genetic information with patient health records are crucial for understanding how genetic variations influence drug response and adverse reactions. Moreover, the heterogeneity of genetic backgrounds among populations further complicates the issue.^79^

Additionally, there are challenges related to data privacy, consent and ethical considerations when it comes to sharing genomic and clinical information.^80^ Striking the right balance between data accessibility and protection of patient privacy is essential but complex. Investments in data infrastructure, standardisation of data formats and protocols and initiatives to promote data sharing and collaboration are critical.

Another challenge is the rapid pace of advancements in PGx, which can make it difficult for healthcare professionals to stay updated with the latest developments.^81^ Without clear guidelines or accreditation standards, healthcare professionals may struggle to identify reputable training opportunities or gauge the quality of the education they receive. Addressing these challenges requires concerted efforts from various stakeholders. Healthcare institutions and professional regulatory bodies can play a crucial role in advocating for the integration of PGx education into medical school curricula, residency training programmes and continuing education courses.^18^

Additionally, there may be barriers to patient acceptability related to trust and confidence in the healthcare system and genetic testing technologies. Patients may have concerns about the privacy and security of their genetic information, as well as apprehensions about potential discrimination or stigmatisation based on genetic predispositions to certain health conditions.^82^,^84^ Commercial companies’ access to patients’ genetic data is also a concern, hence the need for reviewing and updating the existing data privacy act and rules to improve the public preferences towards PGx testing.^66^ Building trust using enhanced medical technologies and addressing these concerns is essential for promoting patient acceptability of PGx testing.^85^ Tailoring educational materials and communication strategies to meet the needs of diverse patient populations is crucial for promoting awareness and acceptability of PGx testing.

PGx testing’s extensive utilisation can reduce healthcare costs and enhance preventive care, population health and community initiatives.^86 87^ Moreover, PGx testing costs have decreased over time, but access for patients may still be restricted by financial issues, especially in primary care settings where resources may be scarce.

## Conclusion

Successful integration of PGx testing into primary care demands a multifaceted approach that strengthens enablers and addresses challenges (online supplemental file 5). This entails enhancing consumer awareness, providing comprehensive training for healthcare providers and furthering scientific research to elucidate both the clinical benefits and cost-effectiveness of such testing. Additionally, it is imperative to conduct feasibility studies encompassing various countries and healthcare systems to fully understand the potential enablers and challenges of implementing PGx testing in primary care. Currently, the available data predominantly stems from the global north, leading to a gap in knowledge regarding its applicability in diverse cultural and resource-constrained settings.

Addressing the high costs associated with PGx testing requires a multifaceted approach. Efforts are needed to streamline testing processes, improve efficiency and reduce the overall cost of testing. This may involve the development of standardised testing protocols, the use of automation and high-throughput technologies and the optimisation of bioinformatics pipelines.

## supplementary material

10.1136/bmjopen-2024-087064 online supplemental file 1

## Data Availability

Data are available upon reasonable request.
