# Supplementary material for: Scoping review of enablers and challenges of implementing pharmacogenomics testing in the primary care settings
Source: BMJ Open. 2024 Nov 5;14(11):e087064. doi: 10.1136/bmjopen-2024-087064 (PMC11552560; doi:10.1136/bmjopen-2024-087064)

Supplementary File 1:

Search strategy for all database:

1. primary care.mp. or exp Primary Health Care
2. exp Pharmacogenomic Variants/ or exp Pharmacogenomic Testing/ or pharmacogenomic*.mp. or genomics/ or exp pharmacogenetics/ or exp pharmaco-omics/ or exp pharmacogenetics/ or *Genome, Human/ or Genomic medicine.mp. or exp Genomic Medicine/ or exp Precision Medicine/ or exp Pharmacogenetics/ or *Genomics/
3. 1 AND 2

Supplementary File 2:

Stakeholders' views and involvement, enablers, and challenges of implementing PGx testing

Study ID	Study type, year	Disease/ Condition under study	Aims/Objectives	Key stakeholders	Country	Key Findings
**Ahmed 2022**	Retrospective study, 2021	Autism	Assess the prescription pattern of 92 psychotropic drugs in autistic patients and measure its pharmacogenomic testing implication.	Physician	Canada	• One third of the psychotropic drugs has a PGx based treatment guideline. Sertraline, citalopram, risperidone and amitriptyline were mostly benefited from PGx testing.<br>• PGx interpretations varied by ethnicity
**Arwood 2020**	2020	Patients in the general internal medicine	A pharmacist-initiated pharmacogenomics clinic and state its success and challenges that came across within two years of its implementation	Pharmacist	United States	• In two years, 91 patients were seen in clinic. Of patients who received PGx, 77% had at least one CYP2C19 and/or CYP2D6 phenotype that would make conventional prescribing unfavorable. Recommendations to physicians was made for 59% of patients; 87% were accepted.<br>• Challenges included PGx reimbursement and referral maintenance.
**Bank 2019**	Prospective multicenter observational study, 2019	Adult patients with an incident prescription for at least 28 days for amitriptyline, atomoxetine, atorvastatin, (es)citalopram, clomipramine, doxepin, nortriptyline, simvastatin or venlafaxine	Assess the feasibility of pharmacist-initiated pharmacogenomic analysis in primary care and investigate the actionable phenotypes for improving patient clinical outcomes.	Community Pharmacist	Netherlands	• Included 200 patients: 90% carried at least one actionable PGx test result. In 31.0% of the incident prescriptions a combination between a drug with a known gene-drug interaction and an actionable genotype was present and a therapeutic recommendation was provided. Recommendations were accepted by clinicians in 88.7% of the patients.<br>• Limited patient accessibility to PGx services. No financial benefit for the involved healthcare professional. Evidence constraints with the implementation of preemptive PGx panel approach in primary care of the European medical sector.

**Bank 2019**	2016	All prescriptions for the selected 45 drugs	To estimate the potential impact of the implementation of pharmacogenetic screening for eight genes related to drugs used in primary care.	Pharmacists	Netherlands	• In 23.6% of all new prescriptions of 45 drugs (n = 856,002 new prescriptions/year), an actionable gene-drug interaction was present.<br>• These GDIs would result in a dose adjustment or switch to another drug in 5.4% of all new prescriptions.<br>• Dispensing Database: Lack of complete clinical data (such as comorbidities, reduced clearance of drugs, and information on indications) in the available dataset.<br>• Lack of data supply to the database by the outpatient pharmacy which often dispense more specialized pharmacotherapy.
**Behr 2023**	25-question survey, 2023	Pain management	To assess clinician knowledge with clinical pharmacogenomic (PGx) scenarios involving commonly used drugs that have both CPIC guidelines and FDA PGx dosing recommendations.	Physicians, physician assistants, and nurse practitioners	United States	• Thirty-four clinicians completed the survey.<br>• Respondents had minimal experience with PGx and limited awareness of PGx resources. Although respondents expressed belief that PGx has utility to improve medication-related patient outcomes, many lack confidence to apply PGx results<br>• to their practice. For clinical drug–gene questions relevant to primary care and/or pain management, respondents scored poorly.
**Bishop 2021**	Commentary, 2021	Mental health	To comment on the role of pharmacists in pharmacogenomics practice	Clinician, Pharmacist	United States	• PGx testing has the potential to optimise antidepressant treatment by tailoring drug choice and reducing treatment failures/occurrence of adverse drug reactions.<br>• Involving pharmacists in the PGx process can leverage their expertise in medication management and patient communication, enhancing the overall effectiveness of PGx implementation.<br>• PGx test results can be complex and difficult to interpret, requiring specialized knowledge and training for clinicians.<br>• Other challenges include variability in PGx tests, lack of clear guidelines on how PGx results should be used in clinical practice, limited evidence base for PGx use in

						mental health, expensive cost of PGx testing, time constraints in primary care
**Biswas 2020**	Case study, 2020	Paediatric Condition	To propose a practical and centralized approach to providing genomic services through an independent, enterprise-wide clinical service model.	Clinician	United States	• Challenges in PGx testing: Lack of knowledge and access to genetics specialists, difficulty interpreting complex test results, insurance reimbursement limitations, integrating genomic findings into patient care.<br>• The Roberts Individualized Medical Genetics Center (RIMGC) Model offers a centralized resource for all clinical divisions, provides services like test selection, insurance pre-authorization, genetic counseling, and result interpretation, collaborates with the diagnostic laboratory for clinical correlation of findings, utilizes "genetic champions" from various specialties for expert input.
**Brown 2017**	A Subanalysis of a prospective trail - 2017	Mental illness	To determine potential cost savings of combinatorial pharmacogenomics testing over one year in patients with mental illness treated by primary care providers and psychiatrists who had switched or added a new psychiatric medication after patients failed to respond to monotherapy.	Primary care providers treat psychiatric patients through general practice, internal medicine, family medicine, and obstetrician/gynecology. Psychiatrist (not included as PCPs)	United States	• Primary care providers (PCPs) congruent with combinatorial PGx testing provided the most medication cost savings for payers and patients at \$3988 per member per year (P < 0.001).<br>• PCPs congruent with the combinatorial PGx test recommendations saved patients \$2690 in medication costs compared with psychiatrists.
**Brown 2021**	Cross-sectional study, 2021	Pediatric patients	Determining availability, concerns, and barriers of pharmacogenomic	Pharmacist, Physician	United States	• Healthcare sector can link the drug gene interaction reports to the clinical decision support of the electronic prescribing system. The most common drug gene interaction test identified in pediatric setting were

			testing in pediatric hospitals			thiopurine/TMPT followed by Voriconazole/ CYP2C19 and Codeine/CYP2D6<br>• Barriers: Cost or reimbursement for the PGx test, potential for genetic discrimination, sharing results with family members, and availability of tests in certified laboratories.
**Brown-Johnson 2021**	Mixed methods research in Quality Improvement, 2021	Patients with cardiovascular risk factors	To assess the implementation outcomes, specifically penetration/reach, acceptability, feasibility, and sustainability of Humanwide, a pilot embedding multi-faceted precision health into a team-based primacy care setting<br>To inform future implementation initiatives and facilitate the scale/spread of precision health in primary care.<br>To assess its early potential clinical benefit to patients.	MDs, Advance Practice Provider (NP or PA) health professionals, diabetes pharmacists, dieticians, mental health providers, triage nurse	United States	• Patients and providers reported Humanwide was acceptable; it engaged patients holistically, supported faster medication titration, and strengthened patient-provider relationships. All patients benefited clinically from at least one Humanwide component.<br>• Feasibility challenges included: low provider self-efficacy for interpreting genetics and pharmacogenomics; difficulties with data integration; patient technology challenges; and additional staffing needs. Patient financial burden concerns surfaced with respect to sustainability.
**Brunette 2019**	Pragmatic Clinical Trial, 2019	Cardiovascular disease (needing statin therapy without previous history of statin use).	To apply Pragmatic Clinical Trial (PCT) principles to The Integrating Pharmacogenetics In Clinical Care (I-PICC) Study.	Primary care provider	United States	• The trial achieved high engagement with providers (85% enrolled of those approached) and enrolled a representative sample of participants for which statin therapy would be recommended.<br>• PCT is a valuable tool for generating high quality and generalizable evidence about the effectiveness of genomic interventions.

			To generate evidence for the clinical utility of pre-emptive pharmacogenetic testing in the initiation of statin therapy.			• PCTs allow for the post-trial implementation of their interventions, increasing the likelihood that beneficial interventions will be taken up into clinical care.<br>• Barriers: Time and resource constraints: Implementing a new testing and intervention process requires additional time and resources from healthcare providers; Patient engagement: Ensuring patient understanding and consent for genetic testing can be time-consuming; Insurance authorization: Obtaining insurance approval for genetic tests can be complex and time-consuming.
**Carroll 2016**	A qualitative study involving focus groups	Cancer	To assess primary care providers' (PCPs) experiences with, perceptions of, and desired role in personalised medicine, with a focus on cancer.	primary care providers	Canada	• Primary care providers have limited experience in personalised medicine; main areas of involvement are breast cancer and prenatal care. PCPs expect growing involvement in personalised medicine due to patient demand and trust<br>• PCPs were concerned over their lack of knowledge, with some who based their practices on personal experiences rather than evidence. They are also concerned about information overload due to the rapid pace of discoveries in geneomics (particularly in direct-to-consumer personal genomic testing).<br>• Need for support: Increased knowledge, collaboration with genetics specialists, and accessible resources are crucial for successful implementation.
**Carroll 2019**	Questionnaire Design and Administratio n	NA	to determine family physicians' (FP) current involvement in GM (general medicine), confidence in GM primary care competencies, attitudes regarding the clinical importance of GM, awareness of genetic	Physicians	Canada	• FPs see their role as making appropriate referrals, are somewhat optimistic<br>• about the contribution GM may make to patient care, but express caution about its current clinical benefits.<br>• There is a need for evidence-based educational resources integrated into primary care and improved communication with genetic specialists.

			services, resources required, and suggestions for changes that would enable the integration of GM into practice.			
**Cavallari 2023**	Review of a Muti-centric cohort, 2023	Adult patients with newly initiated drugs stated in the Dutch Pharmacogenomics Working Group guideline	The effect of twelve gene panel pharmacogenomic testing to prevent adverse drug reactions in patients across seven countries	Pharmacist, Physician	United States	• Effective educational strategy and mechanism for returning pharmacogenetic results led to high recommendation acceptance rate by providers.<br>• Adverse drug reactions significantly declined among the actionable genotype patients where treatment recommendations were considered.
**Chapdelaine 2021**	Secondary data analysis, 2021	Geriatric patients without moderate to severe cognitive impairment	Assess the factors of older adults that affect pharmacogenomic testing in primary care	Primary care providers	Canada	• Majority were willing to provide their samples and pay from their pockets for carrying out PGx analysis for an effective treatment.<br>• Age was inversely proportional to the their willingness to provide samples for PGx analysis. Lower level of education affected their willingness to pay for PGx testing
**Crown 2020**	prospective cohort study	Not Mentioned/Not Applicable	Examining the impact of the CPD program on practicing pharmacists' knowledge, readiness and comfort, and ability to implement pharmacogenomics services in their practices	Pharmacists	Canada	• This multi-component CPD program successfully increased pharmacists' knowledge, readiness, and comfort in applying PGx to patient care in the short-term, yet some pharmacists struggled to integrate this new service into their practices.
**Dressler 2019**	This prospective, observational feasibility study was		Assess feasibility and perspectives of pharmacogenetic testing/PGx in rural primary care physician	Physicians	United States	• Prestudy, no PCP had ever ordered a PGx test. Test results demonstrated gene variations in 30% of patients, related to current medications, with PCPs reporting changes to drug management.<br>• PCPs and patients had favorable responses to testing.

	conducted between September 2016 and December 2017		(PCP) practices, when PCPs are trained to interpret/apply results and testing costs are covered			• PCPs were concerned about their lack of expertise, lack of comfort applying results and out-of-pocket expense for their patients/lack of reimbursement<br>• for the test
**Elliott 2017**	prospective, open-label, randomised controlled trial	50 years and older taking or initiating treatment with at least one of fifty-five single-ingredient or six medication combinations (Polypharmacy)	Assessment of clinical impact of pharmacogenetic profiling integrating binary and cumulative drug and gene inter-action warnings on home health polypharmacy patients	Physicians	United States	• Subjects (n = 110) were randomized to pharmacogenetic profiling (n = 57)<br>• PGx reduced re-hospitalisations and emergency department visits at 60 days.<br>• Of the total 124 drug therapy recommendations passed on to clinicians, 96 (77%) were followed.
**Forester 2020**	Post hoc analysis of data from a blinded, randomised controlled trial comparing two active treatment arms.	major depressive disorder (MDD)	Evaluate the clinical utility of combinatorial pharmacogenomic testing for informing medication selection among older adults who have experienced antidepressant medication failure for major depressive disorder (MDD)	Physicians	United States	• Remission and response rates improved significantly with the use of combinatorial pharmacogenomic testing to identify medications with potential gene-drug interactions and guide medication selection.<br>• At week 8, symptom improvement was not significantly different for guided-care than for treatment as usual (TAU); however, guided-care showed significantly improved response and remission relative to TAU.
**Frigon 2019**	Focus Group interviews/ 2019	NA	To better understand the perceptions of PCPs, pharmacists, and patients regarding the implementation of PGx testing in clinical practice,	Primary care physicians (PCPs), pharmacists and patients	Canada	• Majority of the participants showed enthusiasm toward the implementation of PGx in clinics. The reduction of adverse events is seen as a main benefit of PGx testing.<br>• Challenges: High cost, need for accessible PGx guidelines, ethical (revealing genetic information, confidentiality) and insurance issues, need for training for health professionals, need for computerised systems for successful implementation.

**Gammal 2021**	2021	General population	The problems and solutions concerning the integration of pharmacogenomics to the clinical decision support system in a clinical setting	Physician, Pharmacist	United States	• Integrating pharmacogenomics into electronic health records with customized clinical decision support system requires significant resources and specifically trained personnel to implement and maintain.<br>• Problems: A single pharmacogenomic result can affect various medications; no standard location for pharmacogenomic results in EHR; results should be accessible to all clinicians, like drug allergies; pharmacogenomic results need permanent access, not archiving; result variability: Multiple tests for the same gene can produce different results; evolving evidence: pharmacogenomic interpretations may change over time.<br>• Solutions: Problem list entries: use standardised phenotype terms for actionable pharmacogenomic results; utilize existing drug allergy alerts for high-risk pharmacogenomic findings; train clinicians on the importance of these entries and how to use them; improve data sharing between healthcare institutions; educate patients about their pharmacogenomic results and encourage sharing; promote broader pharmacogenomics knowledge among clinicians; incorporate pharmacogenomic inquiries into standard patient care.
**Grant 2009**	Cross-sectional, 2009	Type 2 diabetes mellitus	Assess the physicians and patient's views on pharmacogenomic testing for the prediction and management of diabetes.	Physicians	United States	• More specialized physicians were more enthusiastic in FDA approved genetic testing for guiding the treatment for diabetes and also predicting the disease. Patients were in more eager for a genetic test that would gain them the best treatment.<br>• Patients were concerned about their privacy, high cost of PGx testing
**Haga 2012**	Cross-sectional Survey & 2012	NA	To seek PCPs views on their willingness and readiness to utilise PGx testing, desirable test properties, and factors	Primary Care Physicians (PCPs)	United States	• Most respondents were aware of PGx testing and recognised its potential to predict drug response. However, few felt confident ordering these tests, and many lacked PGx education.

			relevant to the use of PGx tests			• The majority of respondents felt primarily responsible for informing patients about PGx tests for prescribed medications and deciding how to document PGx results. There was limited recognition of other healthcare professionals' roles in PGx testing, except for disease specialists.
**Haga 2012**	Pilot Study, 2012	NA	To assess attitudes toward PGx testing, ancillary disease risk information, and related clinical issues, we conducted a series of focus groups among health professionals.	Primary care Professionals and Genetic Professionals	United States	• Primary care physicians (PCPs) expressed general interest in pharmacogenomics (PGx) testing but had reservations about its practical application. Concerns included uncertain clinical benefits, insurance reimbursement challenges, potential treatment delays, and difficulties in communicating and interpreting ancillary genetic risks.<br>• While PCPs felt a duty to disclose potential genetic risks to patients, geneticists believe it is not always necessary, emphasizing the complexity of genetic information, such as incomplete penetrance<br>• To optimise the use of PGx testing, expanded educational programs, increased access to genetic experts, and clear clinical guidelines are essential.
**Haga 2014**	2014	General	Displays delivery models of pharmacogenomic screening for healthcare settings	Pharmacist	United States	• Current prescription-driven and pre-emptive PGx models are insufficient for widespread adoption, necessitating alternative delivery strategies.<br>• Incorporating PGx into wellness programs, retail clinics, and whole-genome sequencing offers potential avenues for broader access and utilization.<br>• It is crucial to develop strategies that make testing more accessible and affordable to the general population.
**Haga 2017**	Pilot study, 2017		To investigate provider utilization of pharmacist support in the delivery of pharmacogenetic testing in a primary care setting.	Primary care providers' and Pharmacists.	United States	• Two primary care clinics participated in the study. One clinic was provided with an in-house pharmacist and the second clinic had an on-call pharmacist.<br>• The pharmacogenetic (PGx) training was well-received by most providers, who felt it equipped them to order and utilize PGx tests effectively. Providers with direct access to a pharmacist (in-house) were more likely to

						order PGx tests and consult with the pharmacist compared to those with on-call pharmacist support.<br>• Despite abnormal test results in a third of patients, only a small proportion of drug changes were made. While the in-house pharmacist model showed initial promise, long-term test utilisation was inconsistent. There is a need to explore potential barriers such as insurance, time constraints, or lack of in-house testing facilities.
**Hajek 2022**	2022	NA	To offer guidance to health systems developing genetic education programs that are appropriate to the needs of providers who are not genetic specialists.	Health Care Providers'	United States	• A 2-year genetics education program with quarterly web-based modules that were mandatory for all physicians and advanced practice providers was developed.<br>• The training was effective and boosted healthcare providers' confidence in their genetic knowledge and ability to use genetics.<br>• This demonstrates the potential of scalable digital education to enhance provider readiness in genomic medicine.
**Herman 2014**	Clinical trial, 2014	Non-diabetes patient under evaluation for obstructive coronary artery disease (CAD)	Assessing the benefits of gene expression score in the diagnosis of obstructive CAD	Physicians, nurses, and physician assistants	United States	• The Gene Expression Score (GES) effectively identifies patients without obstructive coronary artery disease (CAD), allowing for faster diagnosis and treatment of non-cardiac causes of chest pain.<br>• Implementing GES in primary care can improve patient care by streamlining the diagnostic process and reducing unnecessary tests for low to intermediate-risk patients, especially women.
**Hundert mark 2020**	The thirteen-question survey, 2020	Pharmacist Knowledge from postgraduate education and training.	The primary objective of this survey was to determine how postgraduate education and training influence pharmacists' knowledge and attitudes toward	Pharmacist	United States	• Pharmacists with post graduate education were more likely to received formal training on PGx, self-rated their knowledge higher, and respond favorably to PGx being offered thorugh pharmacy services. Pharmacists with board certifications were more comfortable interpreting PGx results.<br>• To effectively implement pharmacogenomic testing, leveraging pharmacists with postgraduate qualifications is recommended as a foundational step.

			pharmacogenomic testing.			Comprehensive educational initiatives are essential to equip all pharmacists with the necessary knowledge and skills.
**Hutchcraft 2022**	Single institution prospective cohort study, 2022.	Hereditary Disease	To assess the clinical utility of germline medical exome sequencing in patients recruited from a family medicine clinic and comparing the mutation frequency of hereditary predisposition genes to established general population frequencies.	Physicians	United States	• Germline genetic screening identified hereditary disease predispositions and actionable pharmacogenomic variants in patients.<br>• While pharmacogenomic testing led to medication changes in a small number of cases, the study demonstrated the feasibility of integrating genetic screening into primary care.<br>• Long-term integration of pharmacogenomic test results into electronic health records is crucial to maximize patient benefits.
**Jablonski 2020**	Sub analysis of a 1-year prospective Assessment of medication cost, 2019.	Psychiatric (Mental Illness).	Comparison of economic outcomes when elderly patients with neuropsychiatric disorders received psychotropic medications guided by a combinatorial pharmacogenomic (PGx) test.	Primary Care Providers'	United States	• Aligning medication with pharmacogenomic test results (congruent prescribing) significantly reduced annual drug costs for patients with neuropsychiatric disorders, especially in those aged 65 and older.<br>• Congruent prescribing was associated with a reduction in the number of neuropsychiatric medications for older patients.
**Jarvis 2022**	Retrospective study, 20233	Older adult population	Evaluating a large real-world pharmacogenomic implementation to the comprehensive medication management system in the US	Pharmacist	United States	• A pharmacogenomics-enriched comprehensive medication management program reduced direct medical charges by approximately $7000 per patient (≥65 years) who are receiving benefits through a state retirement system over the first 32 months of a voluntary PGx-enriched comprehensive medication management program.<br>• The program shifted healthcare resource utilization from acute care to primary care.

						• Medication risk assessment, patient-provider communication, and sustained positive healthcare trends support the program's effectiveness.
**Kehr 2023**	Single center, non-interventional, retrospective cohort study.	Older adults within an outpatient geriatric clinic.	The primary objective was to identify the proportion of patients who completed PGx testing. Secondary objectives included determining the proportion of patients with actionable PGx results, determining the proportion of patients with a baseline medication intervention within six months of completing PGx testing, and identifying barriers to not completing testing.	Pharmacist	United States	• Of 67 patients, 72% successfully completed PGx testing, with 72% having actionable PGx findings and 83% having a pharmacological intervention made thereafter.<br>• Nineteen patients did not complete testing (28%), with the primary barrier being not having an appointment scheduled (63%).
**Kennedy 2013**	2013	Psychiatric patients	Feasibility of pharmacogenomic testing in primary care	Physician	Canada	• The integration of PGx reports for CYP450 variants has been well-received by both physicians and patients.<br>• Successful integration of pharmacogenomic (PGx) testing for antidepressants and antipsychotics in primary care.<br>• Demonstrated feasibility of delivering understandable and actionable PGx information to primary care providers.<br>• Anticipated improved treatment outcomes through early-stage PGx testing.
**Kimpton 2019**	Retrospective study, 2019.	Exposure of patients to pharmacogenomic drugs retrospectively.	To investigate the longitudinal exposure of English primary care patients to pharmacogenomic	Practitioners	United Kingdom	• In English primary care, it's highly common for patients to be exposed to multiple pharmacogenomic drugs, with 60% receiving two or more and 18% receiving five or more over 20 years.

			drugs to inform the design of pre-emptive testing.			• Exposure to these drugs typically begins in early adulthood and increases with age.<br>• Three pharmacogenes are responsible for over 95% of the prescribed pharmacogenomic drugs.<br>• There is a lack of evidence on the clinical utility of PGx<br>• These insights could guide the development of pre-emptive pharmacogenomic testing strategies for primary care.
**Ladapo 2015**	Prospective Muti-centric Observational Study, 2015	Coronary artery disease (CAD)	Assess the usage of blood gene expression diagnostic tests and their clinical benefit in confirming obstructive CAD in primary care.	Physician, nurse, phlebotomist, office manager	United States	• A personalized gene expression score (GES) significantly influenced primary care providers' cardiac referral decisions for patients with stable, nonacute chest pain.<br>• Patients with a low GES had a reduced likelihood of being referred for cardiac evaluationcompared to those with elevated GES.
**Leger 2016**	Retrospective study, 2016	HIV infection	Examination of genetic data with the efavirenz discontinuation from central nervous system adverse events in HIV primary care patients of Southeastern United States	Physician	United States	• Among 563 patients, 17.5% discontinued efavirenz within 12 months, with 5.1% stopping due to CNS symptoms.<br>• Slow metabolizers had a significantly higher risk of discontinuing efavirenz for CNS symptoms.<br>• The risk was notably stronger in Whites compared to Blacks.
**Lemke 2017**	Descriptive Study	NA	To explore primary care physicians, views of the utility and delivery of direct access to pharmacogenomics (PGx) testing in a community health system.	Primary Care Physicians	United States	• Benefits of PGx testing include reducing side effects, faster dose titration, enhanced shared decision-making, and offering psychological reassurance.<br>• Challenges to address include privacy concerns, cost, insurance coverage, and the complexity of interpreting PGx test results.
**Li 2014**	Pilot Study, 2014.	Hyperlipidemia (Statin Therapy).	To improve statin adherence, it is tailored to an individuals’ SLCO1B1*5	Physicians	United States	• Sharing pharmacogenetic test results with both patients and healthcare providers can influence medication adherence positively.

			genotype and addresses a major driver of statin adherence in the primary care population.			• This is achieved by increasing patients' understanding of their condition, alleviating medication concerns, and promoting collaborative decision-making.<br>• Delivering SLCO1B1*5 results and recommendations through electronic medical records (EMR) is feasible in a primary care setting.
**Luke 2021**	Qualitative Descriptive Approach, 2021.	In this study, additional internal factors related to the capabilities, opportunities, and motivations of pharmacists that influence their ability to implement PGx testing were analyzed.	To further elucidate the factors influencing the integration of PGx testing by pharmacists in their practices, the BCW approach should be used to inform future intervention options to support pharmacists with this integration.	Pharmacists	Canada	• Pharmacists' professional identities, practice environments, self-confidence, and beliefs in PGx benefits influenced their ability to provide PGx-testing services. Potential interventions to enhance implementation include preparing pharmacists for higher patient volumes, assisting with software and technology navigation, and streamlining workflows and documentation.
**Marzuillo 2014**	A cross-sectional survey, 2014.	A self-administered questionnaire was used to carry out a cross-sectional survey of a random sample of Italian public health professionals.	To assess the knowledge, attitudes, and training needs of public health professionals in the field of predictive genetic testing for chronic diseases.	Public health practitioners	Italy	• Italian public health professionals have a positive attitude toward predictive genetic testing for chronic diseases but require additional training to enhance their methodological knowledge.<br>• Knowledge increases with exposure to genetic testing during postgraduate training, continued medical education, and proficiency in English.<br>• Adequate knowledge strongly predicts positive attitudes toward genetic testing from a public health perspective.<br>• Physicians have lower knowledge levels but more public health-oriented attitudes compared to other professionals.
**Massart 2022**	2022	Public	Describe a precision medicine center using a multi-disciplinary care model in primary care settings	Physicians and pharmacists trained in genetics and	United States	• The clinic includes a primary care physician trained in genetics, a pharmacogenomics-specialized pharmacist, and two genetic counselors.<br>• The clinic accepts referrals, conducts genetic and pharmacogenomic testing, and provides follow-up

				genetic counselors		care, with results and care plans shared back with referring clinicians.<br>• Since its launch, the clinic has received 99 referrals, demonstrating the model's success in expanding access to genetic services and increasing clinician collaboration and awareness.<br>• This innovative model may serve as a template for other health systems looking to offer precision medicine services in primary care.
**Mills 2013**	2013	Public	Key elements to communicate with patients before and when reporting pharmacogenomic data	Physician, pharmacist, and genetic counselor	United States	• Challenges: Slow adoption due to unclear guidelines on who should order tests, when to order, and how to communicate results, combined with PCPs' limited familiarity with PGx testing.<br>• Patient Preferences: Patients prefer receiving PGx results from trusted PCPs.<br>• Pre-Test Communication: Key topics include the purpose of the test, risks/benefits, the genetic basis of PGx testing, and its future benefits for other treatments.<br>• Post-Test Communication: Focus on clear communication of results, implications for future treatments, and providing summary letters or referrals as needed.
**Mwale 2021**	Qualitative interview/Semi-structured interviews with GPs as well as documentary analysis of policy/ 2021	N/A<br>Genomic medicine in the NHS and practice implementation	To explore GPs, views on mainstreaming genomic medicine in the NHS and implications for their practice.<br><br>To examine how visions of genomic futures in the NHS are conceived and received by<br>GPs by engaging the	General practitioners (GPs)	United Kingdom	• Facilitators for PGx Implementation: policy documents present a positive vision of genomic medicine as a transformative technology, indicating its potential to improve diagnosis and treatment within the NHS; genomic medicine is seen as capable of providing personalized treatments and identifying genetic determinants of diseases, which can enhance patient care.<br>• Barriers: many general practitioners (GPs) feel inadequately informed about genomics and its implications for clinical practice, resulting in skepticism regarding its relevance and applicability; current healthcare infrastructure lacks the necessary systems

			concept of "sociotechnical imaginaries."<br><br>To undertake documentary analysis of publicly available policy documents relating to the mainstreaming of genomics, such as the Human Genomics Strategy Group (2012), the Chief Medical Officer of England's (2016) report, the Life Sciences Industrial Strategy (2020), and editorial material on NHSE and Genomics England websites provided an alternative official account of how genomic futures are imagined, presented, and enacted.			to effectively integrate genomic medicine into everyday clinical practice, hindering its implementation; GPs prioritize pressing patient care needs over genomic initiatives, viewing genomics as a low priority in light of existing challenges within primary care; concerns about the complexities of genomic testing and its implications for patient expectations create anxiety among GPs, leading to reluctance in adopting genomics as a routine practice.
**Natasha Petry 2019**	The five Iâ€™sâ€™ as a template for other institutions seeking to start a "de novo" pharmacogen	Manuscript, 2019.	Describes our efforts to place pharmacogenomics in the hands of the primary care provider, integrating this information into a patient's healthcare over their lifetime.	Pharmacists, Nurses, Genetic Counselors, and other healthcare workers	United States	• Facilitator: A multidisciplinary team, including pharmacists, genetic counselors, and lab scientists, collaborates to integrate PGx into primary care. This team approach is supported by automated decision support systems that provide real-time alerts and recommendations based on established guidelines, helping healthcare providers make informed prescribing decisions for patients based on their genetic profiles.

	omics program.					• Barrier: Despite the advantages of PGx testing, limited provider knowledge about PGx remains a significant challenge. Many healthcare professionals lack adequate training in PGx, leading to difficulties in interpreting test results and implementing recommendations in clinical practice. Additionally, standardizing PGx testing processes and integrating them into electronic medical records (EMRs) pose operational challenges that can impede the widespread adoption of these personalized medicine approaches across healthcare systems.
**O'Donnell 2017**	Prospective	NA	To examine prospectively the impact of available pharmacogenomic information on physician prescribing behaviors.	Physicians	United States	• The clinical decision support (CDS) system utilized traffic light alerts (green for favorable, yellow for caution, and red for high risk) to communicate pharmacogenomic information to providers.<br>• Analysis of 2,279 outpatient encounters showed that medications classified as high pharmacogenomic risk were changed significantly more often than those without such information.<br>• Medications with cautionary pharmacogenomic information were also changed more frequently.<br>• Improved decision-making to reduce patient risk through the integration of genomic medicine into clinical practice.
**O'Shea 2022**	A questionnaire study, 2022.	An anonymous, online questionnaire generated using Qualtrics® and circulated via social media and posters placed in eight participating community pharmacies was	To establish perceptions of pharmacogenomics (awareness, understanding, openness to availability, perceived benefits and concerns, willingness to pay, and service setting) and investigate if they differ between those	Community Pharmacists, Primary Healthcare Providers	Ireland	• Low awareness and knowledge of pharmacogenomics among the general population.<br>• After being informed about pharmacogenomics, patients with chronic diseases were 2.17 times more likely to desire the availability of pharmacogenomic services compared to those without chronic conditions<br>• Willingness to pay for pharmacogenomic testing was not influenced by chronic disease status.<br>• Respondents preferred pharmacogenomic services to be offered in primary care settings rather than hospitals.

		conducted with Irish adults.	with and without chronic disease(s).			
**Olander 2018**	Survey, 2018.	NA	The primary objective of this survey was to ascertain primary care clinicians' perceptions of pharmacogenetic use and implementation in an integrated health system of metropolitan and rural settings across several states.	Primary Care Clinicians	United States	• Of the 90 respondents, (90%) of respondents felt uncomfortable ordering pharmacogenetic tests, and 76% were uneasy about applying the test results in clinical practice.<br>• 78% of respondents expressed interest in having pharmacogenetic testing available through Medication Therapy Management (MTM) services, although physician assistants showed less interest compared to nurse practitioners and medical doctors/doctors of osteopathy.<br>• 95% of respondents indicated interest in a clinical decision support tool related to pharmacogenetic results.<br>• Overall, primary care clinicians are hesitant to engage with pharmacogenetics; however, the positive attitude towards incorporating testing into MTM services presents an opportunity for pharmacists to enhance their practices.
**Olson 2017**	A prospective, randomised study	Neuropsychiatric Disorders	Pharmacogenetic testing holds promise as a personalised medicine tool by permitting individualization of pharmacotherapy in accordance with genes influencing therapeutic response, side effects, and adverse events. The authors evaluated the effect of outcomes for the patients diagnosed with neuropsychiatric	Clinicians	United States	• A prospective, randomized study was conducted with 237 patients at a community-based psychiatric practice, comparing PGx guided treatment with standard care.<br>• More than half (53%) of patients in the control group experienced at least one adverse drug event, while only 28% of patients receiving PGx-guided medication management reported adverse events (P = .001).<br>• Both groups showed improvements Neuropsychiatric Questionnaire (NPQ) and Symbol Digit Coding Test (SDC) scores, but no statistical difference.<br>• Pharmacogenetic testing can enhance the tolerability of psychiatric drug therapy while maintaining similar efficacy compared to standard treatment.

			disorders of pharmacogenetics-guided treatment compared to the usual standard of care.			
**Overkleeft 2020**	A Bioinformatics Approach, 2020.	The illustration of the 4MedBOX system.	To provide a description of the Personal Genetic Locker project and show its utility through a use case based on open standards, which is illustrated by the 4MedBox system.	Primary care professionals	Netherlands	• Facilitators: The Personal Genetic Locker (PGL) Project provides an ICT infrastructure for individuals to access and manage their genetic health data, enhancing personalized medicine. This includes clinical decision support systems that aid clinicians in treatment decisions, collaborative development with partners like 4MedBox, and a focus on establishing a strong ethical foundation to address the implications of genetic data use.<br>• Barriers: The implementation of pharmacogenomics faces challenges such as the lack of clear guidelines for translating test results into clinical actions, trust issues regarding the reliability of non-standard genetic data, and the need for specialized training for healthcare providers. Additionally, ethical and legal concerns about consent and privacy must be addressed, alongside technological hurdles for data sharing and a need for greater public awareness of genetic research.
**Papastergiou 2017**	Open-label, non-randomised, Observational.	NA	To evaluate the feasibility of implementing personalised medication services into community pharmacy practice<br>To assess the number of drug therapy problems identified as a result of pharmacogenomic screening	Pharmacists	Canada	• Pharmacists offered PGx screening as part of their professional services program.<br>• A total of 100 patients participated in the program.<br>• Common reasons for pharmacogenomic testing included ineffective therapy (43.0%), addressing adverse reactions (32.6%), and guiding therapy initiation (10.4%).<br>• An average of 1.3 drug therapy problems related to pharmacogenomic testing were identified per patient, leading to pharmacist recommendations such as therapy changes (60.3%), dose adjustments (13.2%), drug discontinuations (4.4%), and increased monitoring (22.1%).

						• The study demonstrates community pharmacists' readiness to adopt pharmacogenomic screening, enabling them to enhance medication therapy management and provide personalized medication services.
**Papastergiou 2021**	Prospective, single-blind, randomised controlled design	Major depressive disorder and/or generalized anxiety disorder,	Impact of pharmacogenomics guided versus standard antidepressant treatment of depression and anxiety, implemented in three large community pharmacies.	Pharmacists	Canada	• 213 outpatients diagnosed with major depressive disorder and/or generalized anxiety disorder were randomized to receive either pharmacogenomics-guided treatment (n = 105) or standard antidepressant treatment (n = 108).<br>• Participants receiving PGx-guided treatment demonstrated greater improvements in the primary outcome (depression) and two secondary outcomes (generalized anxiety and disability).<br>• Treatment satisfaction improved similarly in both groups
**Park 2007**	Focus group Interviews	Smoking Cessation /Tobacco dependence	(a) to explore physicians' attitudes toward treatment strategies that include matching patients to smoking cessation treatment by genotype, and (b) to identify concerns that would need to be addressed prior to the clinical integration of a genetic test to tailor smoking cessation treatment.	Physicians	United States	• Physicians recognized the potential of genetically tailored treatment to improve smoking cessation efforts for patients trying to quit.<br>• Several barriers to clinical integration were noted, including: misunderstandings by patients about the implications of genetic test results; potential misinterpretation of information related to racial differences in the prevalence of certain risk alleles; concerns about discrimination against patients undergoing genetic testing.<br>• Physicians expressed heightened concerns when informed that the same genetic markers used for tailoring smoking treatment are also linked to a higher risk of nicotine addiction and other psychiatric disorders.<br>• To effectively integrate genetic testing into routine practice, primary care physicians require additional educational resources and system support.
**Prather 2022**	Case Report/2022	Post CVA (Cerebro Vascular Accident)	Assessing the positive impact of personalised	Pharmacist	United States	• A 71-year-old female of European descent enrolled in a pharmacogenomics-enriched comprehensive

			medicine in post-CVA patients with idiopathic symptoms			medication management (PGx+CMM) program, following a cerebrovascular accident.<br>• The PGx+CMM pharmacist utilized a clinical decision support system (CDSS) to review and adjust the patient's medication regimen, communicating recommendations to the prescribing physician.<br>• Following the adjustments, the patient experienced rapid improvement in symptoms, indicating that they were likely due to medication side effects, while maintaining controlled blood pressure and cholesterol levels.
**Rafi 2020**	A Qualitative Study, 2020.	Semi-structured interviews were undertaken with 18 clinical participants (16 GPs and two other clinicians). All interviews were recorded and transcribed verbatim.	To explore the potential barriers, opportunities, and challenges facing the implementation of pharmacogenomics into primary care.	General practitioners	United Kingdom	• Barriers: Participants expressed concerns about the cost-effectiveness of implementing PGx in primary care, as well as ethical, legal, and social implications associated with the use of genomic information.<br>• Opportunities: The increasing availability of direct-to-consumer testing presents an opportunity to drive awareness and understanding of PGx in primary care, emphasizing the need for education and workforce training.<br>• Challenges: Key challenges identified include the need to educate the primary care workforce on PGx, address the economic and informatics aspects of implementation, and consider the potential impact on patients before integrating genomic testing into routine practice.
**Rigter 2020**	Focus group Interviews, Meetings, and Delphi Technique		To define actions, roles, and responsibilities for the implementation of pharmacogenetics by conducting a multi-phased stakeholder study.	pharmacists and primary care physicians	Netherlands	• Lack of evidence for the clinical utility of PGx was identified as a significant barrier to its integration into primary care.<br>• Reimbursement policies and effective data registration and sharing are crucial for the routine application of PGx.<br>• There is currently a lack of clarity regarding the division of roles and responsibilities between general practitioners and pharmacists in the context of PGx.

						• During an expert meeting, 16 actions were proposed across four areas (clinical utility, reimbursement, data registration and sharing, and roles and responsibilities), with nine actions remaining pertinent after a Delphi Study.<br>• Participants exhibited low agreement on the prioritization of actions, highlighting different perspectives and the need for better alignment among stakeholders.<br>• Effective and efficient implementation of PGx in primary care could be facilitated by coordinating independent initiatives among various stakeholders.
**RodrÃ-guez-Escudero 2020**	Pilot study, following a pre- and post-interventional experimental design, 2020	Psychiatry	aimed at demonstrating the benefit of incorporating PGx information into Comprehensive Medication Management (CMM) services.	Pharmacist	Puerto Rico	• Pharmacists created new Medication Action Plans (MAPs) for each patient based on PGx results, leading to personalized treatment recommendations.<br>• Genetic variants affecting drug safety and effectiveness were identified in 96% of patients, prompting pharmacists to modify initial treatment recommendations.<br>• Polymorphisms in key isoenzyme genes—CYP2D6 (83%), CYP2C19 (52%), and CYP2C9 (41%)—were identified among the patients.<br>• Pharmacists identified 22 additional medication-related problems following PGx determinations, highlighting their role in comprehensive medication management (CMM).
**Schwartz 2017**	2017	Hyperlipidemia<br>Hypertension<br>Type 2 diabetes mellitus<br>Hypothyroidism<br>Vitamin D deficiency<br>Allergic rhinitis<br>Anxiety<br>Gastroesophageal	The purpose of this study was to implement a clinical pharmacist-led MTM service within a primary care setting that is enhanced by 1) a clinical decision support system (CDSS) that includes a unique	Pharmacist	United States	• Patients enrolled in the study used an average of 12.1 (± 4.6) medications.<br>• Average turnaround time for Medication Therapy Management (MTM) Plus consults was 11.7 (± 6.2) days.<br>• Pharmacists identified a total of 138 medication-related problems (MRPs) during the consults.<br>• Most frequent types of MRPs included drug-drug interactions (29.0%) and drug-gene interactions (DGIs; 24.6%).

		reflux disorder<br>Major depressive disorder<br>Insomnia	combination of medication risk mitigation factors, which aids the pharmacist in interpreting the medication profile, and 2) pharmacogenomics (PGx) testing			• Clinical pharmacist-led MTM Plus service in a primary care setting is feasible and effective.<br>• DGIs are prevalent among older adults in family practice, and PGx testing can reveal additional MRPs that might otherwise be overlooked.
**Sharma 2017**	Validation Study, 2017.	Opioid Use Disorder.	To determine the predictability of aberrant behavior to opioids using a comprehensive scoring algorithm incorporating phenotypic and, more uniquely, genotypic risk factors.	Primary care Physicians	United States	• In a validation study involving 452 participants diagnosed with opioid use disorder (OUD) and 1,237 controls, the algorithm demonstrated 91.8% sensitivity in categorizing patients at high and moderate risk for OUD.<br>• The sensitivity of the algorithm remained above 90% even with changes in the prevalence of OUD.<br>• The algorithm effectively stratifies primary care patients into low-, moderate-, and high-risk categories, aiding in the identification of those requiring additional guidance, monitoring, or treatment adjustments.
**Shields 2008**	Survey, 2008	Smoking Cessation	To assess physicians' willingness to offer a new genetic test to tailor smoking treatment individually	Physicians	United States	• Physicians' likelihood of offering a new genetic test for tailoring smoking cessation treatment ranged from 69–78% across scenarios.<br>• Their willingness significantly decreased when informed that the test could identify predisposition to nicotine addiction, differ by race, or have associations with other conditions.<br>• The term "genetic" versus "non-genetic" significantly reduced the likelihood of physicians offering the test in all scenarios.<br>Effective education for primary care physicians is essential for the successful integration of pharmacogenetic strategies for smoking treatment.
**Shields 2008**	2008	Drugs and Alcohol Addiction	To review challenges related to provider readiness.	Physicians	United States	• Key challenges to integrating pharmacogenetics into clinical practice include ensuring primary care physicians' preparedness, patients' willingness to

			To address physicians' knowledge of genetics and the barriers posed by complex genetic traits in particular. To document PCPs' actual experience in ordering and referring patients for genetic testing. Finally, To make recommendations for addressing these concerns and for facilitating the integration of pharmacogenetic treatment strategies for addiction into primary care practice.			undergo testing, the availability of resources and infrastructure, adequate financing and reimbursement, and robust privacy protections to prevent stigmatization and discrimination.<br>• Training in clinical genetics, accurate knowledge of legal protections, and preparedness to counsel patients about genetic testing were all significant predictors for having ordered and/or referred a patient for genetic testing.
**Silva 2021**	Informatic and Bioanalytic method, 2021.	Chronic diseases such as antiepileptic, antiemetics, and antihypertensives.	To provide facile clinical decision support to inform and augment medication management in the primary care setting.	Pharmacists	United States	• PGx examines how individual genes, either alone or in combination with other genetic factors, impact drug responses.<br>• PGx integrates pharmacology and genomics to create personalized, safe drug treatment plans based on an individual's genetic profile.A major challenge in PGx is the absence of comprehensive clinical-genomic databases that can link genotypes, drug dispensing data, and patient outcomes, hampering progress in the field.
**Smith 2022**	Prospective Cohort Study Design, 2022.	The general practitioners recruited 189 patients between October 2020 and March 2021. The	To assess the feasibility of collecting buccal samples by general practitioners (GPs) at private practices in Singapore within a	General practitioners	Singapor e	• Seven GPs from six private practices in Singapore recruited 189 patients for pharmacogenetic testing, with all patients having at least one actionable genetic variant.<br>• The prevalence of patients with two, three, or four variants was 37.0%, 32.8%, and 12.7%, respectively.

		sample size was calculated on the basis of allele frequencies from a similar primary care study in Canada.	usual consultation, incorporating the use of a pharmacogenetics-based medical decision support system to guide subsequent drug dosing.			• Potential medication alterations were identified using a Clinical Decision Support System.<br>• Patients were accepting, and GPs were enthusiastic about the potential of pharmacogenetics to personalize medicine.<br>• The study demonstrated the feasibility of pharmacogenetic testing in primary care
**Srinivasan 2021**	Open-ended, semi-structured interviews, 2021.	Patients who received positive genomic screening results.	To examine primary care providers (PCP) experiences in reporting genomic screening results and integrating those results into patient care.	Primary Care Providers	United States	• Of the 500 patients who underwent genomic screening, 10 received results indicating a genetic variant requiring clinical management.<br>• PCPs valued genomic screening for its benefits to patients and their families and advocated for the inclusion of underrepresented minorities in genomic research.<br>• Challenges identified by providers included maintaining patient contact over time, arranging follow-up care, and managing results with limited genetics expertise.<br>• Ethical concerns were raised about offering genomic sequencing to patients who might not afford diagnostic testing or follow-up care due to financial constraints.
**StSauver 2016**	Survey, 2016.	A total of 159 clinicians within the Mayo Clinic primary care practice received email surveys with the aim of gaining insights into their views regarding the integration and application of pharmacogenomic testing within their clinical practice. These surveys were designed to	To describe early clinician experience with pharmacogenomics in the clinical setting.	Primacy Care Physicians	United States	• Of 90 clinicians, 52% did not expect to use or were unsure about using pharmacogenomic information in future prescribing practices.<br>• 53% found pharmacogenomic alerts confusing, frustrating, or difficult to navigate for additional information.<br>• Only 30% of clinicians who received a CDS alert changed their prescription to an alternative medication.<br>• The study suggests a general lack of clinician comfort with integrating pharmacogenomic data into primary care.

		evaluate the clinicians' sentiments regarding pharmacogenomics and to gauge their opinions on the usefulness of electronic pharmacogenomics clinical decision support (PGx-CDS) alerts.				
**Swen 2012**	Elderly patients over the age of 60, who were on multiple medications and had used at least one drug falling under specific Anatomical Therapeutic Chemical (ATC) codes, including within the previous two years, were chosen randomly for the study, 2012.	Patients were selected from the pharmacy records if they used at least one drug that CYP2D6 metabolizes or CYP2C19 and at least four additional drugs in the preceding two years.	To investigate the feasibility of pharmacy-initiated pharmacogenetic screening in primary care with respect to patient willingness to participate, quality of DNA collection with saliva kits, genotyping, and dispensing data retrieved from the pharmacy.	Pharmacists	Netherlands	• 58.1% of invited patients were willing to participate in the PGx screening study, indicating a high level of acceptance despite the screening not being tied to a specific clinical issue.<br>• Pharmacy-initiated PGx screening is feasible in primary care, but challenges include difficulties in saliva production, particularly for patients on anticholinergic medications, and a 6.7% no-call rate for CYP2D6 on the AmpliChip.

**Tanner 2018**	A naturalistic, open-label, prospective study, 2018.	Major Depressive Disorder, Depression.	To evaluate the utility of combinatorial pharmacogenomics in patients with major depressive disorder in primary care and psychiatric care settings.\ To evaluate symptom improvement, response, and remission rates following treatment guided by combinatorial pharmacogenomic testing among patients with major depressive disorder enrolled in a large, prospective study.	Primary care physicians, psychiatrists	Canada	• A study involving 1,871 patients with Major Depressive Disorder (MDD)<br>• Pharmacogenomic testing categorised medications based on gene-drug interactions, with Beck's Depression Inventory (BDI) scores assessed at baseline and follow-up.<br>• Results showed a 27.9% reduction in depression symptoms, with a 25.7% response rate (≥50% decrease in BDI) and a 15.2% remission rate (BDI ≤10).<br>• Patients treated by primary care providers had significantly better outcomes compared to those treated by psychiatrists, with higher symptom improvement, response, and remission rates.<br>• Patients taking genetically congruent medications (with little or no gene-drug interactions) had a 31% relative improvement in response rate compared to those taking incongruent medications.<br>• The study supports the use of pharmacogenomics in broader treatment settings, particularly in primary care.
**Tiwari 2022**	Rater-blinded, randomised, controlled trial, 2022	Depression	To evaluate the utility of the combinatorial pharmacogenomic test in a Canadian population, this trial was assessed in conjunction with a trial conducted in a U.S. population (GUIDED trial).	physicians	Canada	• Patients in the PGx guided-care arm showed greater symptom improvement (27.6% vs. 22.7%), response (30.3% vs. 22.7%), and remission rates (15.7% vs. 8.3%) compared to treatment as usual, though differences were not statistically significant.<br>• Results suggest that combinatorial PGx testing can be a useful tool for guiding depression treatment within the Canadian healthcare system.
**Turkmen 2023**	The study analyzed up to 32 360 UK Biobank participants	Incident diagnosis of coronary heart disease, heart failure (HF), chronic kidney disease,	To estimate associations between reported pharmacogenetic variants and incident	General Practitioners	United Kingdom	• The study analyzed 32,360 UK Biobank participants prescribed dihydropyridine calcium channel blockers (dCCB) in primary care, focusing on 23 genetic variants.<br>• Key findings include that carriers of the rs877087 T allele in the RYR3 gene had an increased risk of heart

	prescribed dCCB in primary care (from UK general practices, 1990â€ "2017), 2022.	edema, and switching antihypertensive medication.	adverse events in a community-based cohort prescribed dihydropyridine calcium channel blockers.			failure (HF), with a hazard ratio of 1.13, although this was not significant after correction for multiple testing.<br>• If rs877087 T allele carriers experienced the same treatment effect as non-carriers, the incidence of HF could potentially reduce by 9.2%.<br>• Patients with rs10898815 in NUMA1 and rs776746 in CYP3A5 were more likely to switch to an alternative antihypertensive medication.<br>• Other genetic variants studied did not show strong or consistent associations with adverse clinical outcomes.
**vanderWouden 2016**	Longitudinal, prospective cohort study, 2016.	DTC PGT consumers.	To describe the characteristics and perceptions of DTC PGT consumers who discuss their results with their PCP.	Primary Care Providers	United States	• 63% of respondents planned to share their pharmacogenomic results with their primary care provider (PCP), but only 27% did so at 6-month follow-up.<br>• Common reasons for not sharing results included perceiving them as not important enough (40%) or not having time (37%).<br>• Among those who discussed their results with a PCP, 35% were very satisfied, while 18% were not satisfied at all.<br>• Key Encounter Themes: Frequently mentioned themes included the actionability of results (32%), PCP engagement (25%), and lack of PCP engagement (22%).
**vanderWouden 2019**	The prospective pilot study, 2019.	In this study, Community pharmacists were provided the opportunity to request a panel of eight pharmacogenetics to guide drug dispensing within a clinical decision support system (CDSS) for 200	To quantify both the feasibility and the real-world impact of this approach in primary care.	Community pharmacists	Netherlands	• Community pharmacists used a panel of eight pharmacogenes to guide drug dispensing for 200 primary care patients, with follow-up after an average of 2.5 years.<br>• PGx-panel results were recorded in 96% of pharmacist and 68% of general practitioner electronic medical records (EMRs).<br>• 97% of patients reused PGx-panel results for at least one new prescription, with 33% using it for up to four prescriptions.<br>• 24.2% of these prescriptions had actionable drug-gene interactions (DGIs) that required pharmacotherapy adjustments.

		primary care patients.				• No difference in healthcare utilization was observed between patients with and without actionable DGIs.<br>• Pre-emptive panel-based pharmacogenetic testing is feasible and has a substantial real-world impact in primary care.
**vanderWouden 2020**	PREPARE study, 2020.	Enrollment of patients under their pharmacists who plan to initiate one of 39 drugs with a Dutch Pharmacogenetics Working Group (DPWG) recommendations.	To study pharmacists' perceived enablers and barriers for PGx panel-testing among pharmacists participating in a PGx implementation study.	Pharmacists	Netherlands	• Barrier: Unclear procedures for implementing PGx testing; undetermined reimbursement for PGx tests and consultations; insufficient evidence of clinical utility for PGx panel testing; infrastructure inefficiencies affecting implementation; limited knowledge and awareness of pharmacogenetics among healthcare professionals.<br>• Enabler: Pharmacists' perceived role in delivering pharmacogenetics; belief in the clinical utility of pharmacogenetics.<br>• Despite a strong belief in the benefits of pharmacogenetics, existing barriers hinder its implementation in primary care settings.
**Vassy 2018**	Qualitative Analysis, 2018.	Primary Care Physicians and their generally healthy patients undergoing genome sequencing	To illuminate how PCPs communicate different types of genome sequencing results and their management recommendations for those results of uncertain clinical utility.	primary care physicians	United States	• In a study of 48 PCP–patient visits, a "take-home" message (recommendation) was identified for each genomic result discussed, categorized into (1) continuing current management, (2) further treatment, (3) further evaluation, (4) behavior change, (5) remembering for future care, or (6) sharing with family members. Quantitative analysis revealed that continuing current management was the most common recommendation, accounting for 66% of all recommendations. Pharmacogenetics prompted recommendations to remember for future care in 79% of cases, while carrier status led to sharing with family members in 83% of instances.<br>• Polygenic results frequently resulted in behavior change recommendations. For monogenic results, 25% of recommendations were for further evaluation.

						• Rationales for recommendations were based on patient context, family context, and scientific/clinical limitations of sequencing.<br>• Overall, PCPs distinguished substantive differences among categories of genomic sequencing results and tailored their clinical recommendations accordingly.
**Vassy 2020**	Randomised trial, 2020.	Statin myopathy risk.	To determine the impact of delivering SLCO1B1 pharmacogenetic results to physicians on the effectiveness of atherosclerotic cardiovascular disease (ASCVD) prevention (measured by low-density lipoprotein cholesterol [LDL-C] levels) and concordance with prescribing guidelines for statin safety and effectiveness.	Physicians	United States	• The study involved 408 patients randomized into intervention (193 patients) and control (215 patients) groups to assess SLCO1B1 genotype effects on statin therapy.<br>• 120 patients (29%) had a genotype indicating increased simvastatin myopathy risk; statin therapy was offered to 33.7% in the intervention group and 32.1% in the control group.<br>• At 12 months, LDL-C reductions were noninferior between the intervention (-1.1 mg/dL) and control (-2.2 mg/dL) groups, with no significant difference in guideline-concordant statin prescriptions (6.2% vs. 6.5%).<br>• Few documented cases of statin-associated muscle symptoms (SAMS) in both groups.<br>• The findings suggest that reporting SLCO1B1 results did not adversely impact atherosclerotic cardiovascular disease prevention and may have led to avoiding simvastatin prescriptions for genetically at-risk patients
**Weinstein 2020**	A qualitative study, 2019.	Depression	To explore pharmacist and physician perspectives on the utility and critical considerations for designing a pharmacist-run pharmacogenomic service for depression in primary care.	Pharmacists	United States	• Pharmacogenomics can help tailor initial medication choices for patients with depression in primary care.<br>• A pharmacist-driven pharmacogenomics service should start with prescriber-patient interactions and involve a collaborative, team-based approach with effective communication.<br>• Trained pharmacists in partnership with outpatient physician practices are essential for interpreting pharmacogenomic results and recommending appropriate medications.

						• Successful implementation requires careful patient selection, engagement, and education.<br>• Monitoring and follow-up care responsibilities will be shared among team members.<br>• Ongoing education for healthcare professionals on interpreting and implementing pharmacogenomic data in depression treatment is essential.
**Wildin 2022**	Consolidated Framework for Implementation Research (CFIR), 2022.	Genetic Disease.	To review the barriers, solutions, and perceived gaps in the context of an implementation research framework.	Primary Care Provider	United States	• The pilot implementation of clinical genomic population health screening for any-health-status adults demonstrated feasibility, successfully translating prior research into clinical practice by centering primary care and using a clinically relevant gene panel.<br>• Key strengths included engaging leadership, securing buy-in from medical administration, involving diverse stakeholders, and leveraging existing workflows, alongside contracting with a commercial laboratory for testing and reporting.<br>• Indirect measures of success showed continued volunteer participation from new primary care providers (PCPs), ongoing patient testing, and minimal complaints related to process and communication.<br>• Barriers to scaling included underestimating the need for leadership engagement in health information technology (HIT), challenges with electronic health record (EHR) integration, and issues with tracking patient attribution.<br>• Adaptations to the process, such as an EHR-plus-paper order method, increased the burden on clinic staff and contributed to tracked process errors<br>• Resilience was supported by the strong knowledge and experience of the implementation team and continued involvement of patient-focused advocates, despite disruptions like the COVID-19 pandemic and a cyberattack.

**Williams 2016**	Qualitative study, A top-down sampling method, 2016.	Alcohol use disorders	Qualitative interviews with primary care providers from 5 clinics in the Veterans Health Administration (VA) to assess their interest in using a genetic test to inform the treatment of alcohol use disorders with pharmacotherapy.	Primary Care Providers, physicians	United States	• Participants showed general interest in using genetic tests to aid in alcohol use disorder (AUD) treatment planning.<br>• Perceived benefits of pharmacogenetic testing included aiding therapeutic choice and enhancing patient motivation and engagement in treatment.<br>• Perceived drawbacks included potential limitations in pharmacotherapy benefits by narrowing the target population and negative impacts from "negative" test results.<br>• Clinical utility was viewed with caveats, as its effectiveness would depend on prognostic accuracy and medication characteristics.<br>• There was uncertainty about whether the test would influence clinical decision-making.<br>• Pragmatic barriers to implementation included costs and the need for resources such as laboratory facilities.
**Youssef 2021**	A comprehensive analysis of a large community pharmacy database was conducted, in 2021.	A total of 56 drugs with 56 unique drug-gene interactions were included in the study for instance (Warffarin, Zuclopenthixol, Carbamazepine).	To quantitatively estimate the volumes of medicines impacted by the implementation of a population-level, pre-emptive pharmacogenetic screening program for nine genes related to medicines frequently dispensed in primary care in 2019.	Pharmacists	United Kingdom	• Actionable drug-gene interactions (DGI) were present in 19.1% to 21.1% of new prescriptions for these drugs, affecting approximately 5,233,353 to 5,780,595 prescriptions out of a total of 27,411,288 new prescriptions per year.<br>• These actionable DGIs would necessitate increased monitoring, maximum ceiling dose precautions, or changes in drug regimen.<br>• Immediate dose adjustments or changes in medication regimen accounted for 8.6% to 9.1% of the prescriptions with actionable DGIs.<br>• The study highlights the frequent occurrence of actionable DGIs in UK primary care, indicating significant opportunities to optimize prescribing practices.

Supplementary File 3: Key stakeholders for the implementation of pharmacogenomics testing in the primary care settings.

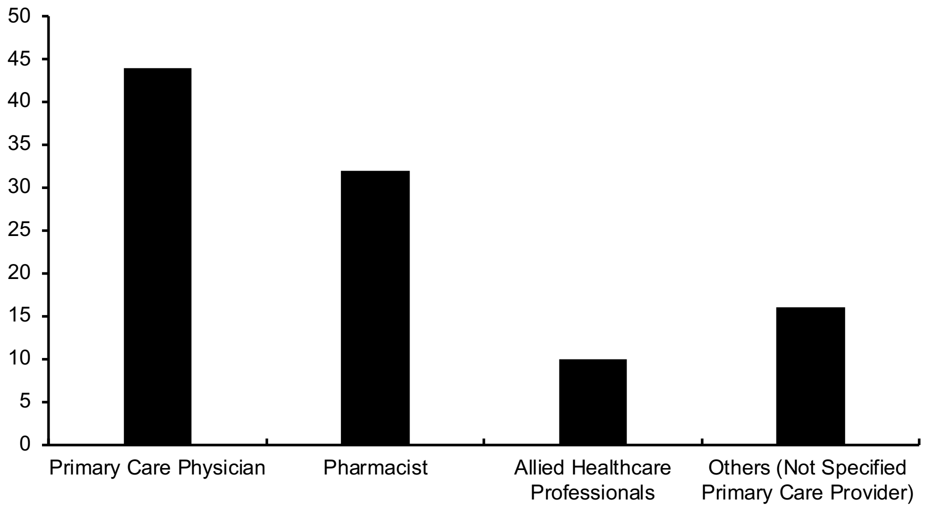

Supplementary File 4: Opinion towards implementation of pharmacogenomics testing in the primary care settings

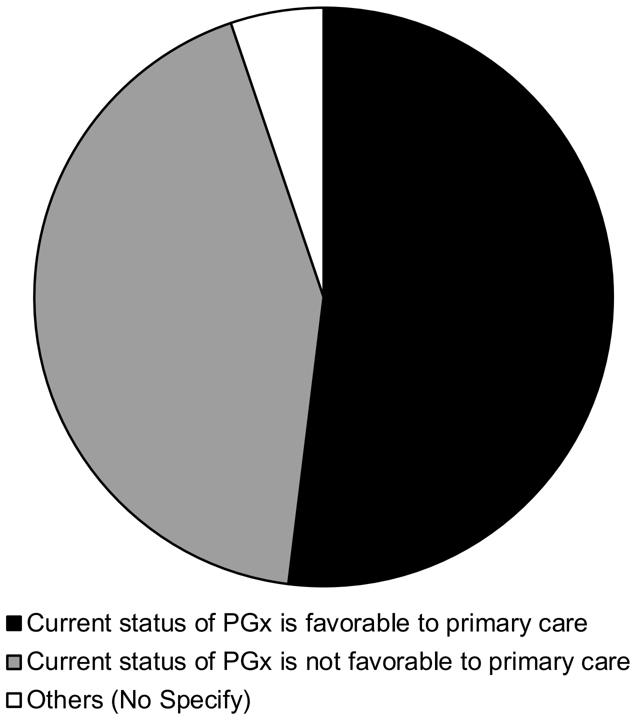

Supplementary File 5: Enablers and challenges of implementation of pharmacogenomics testing in primary care settings

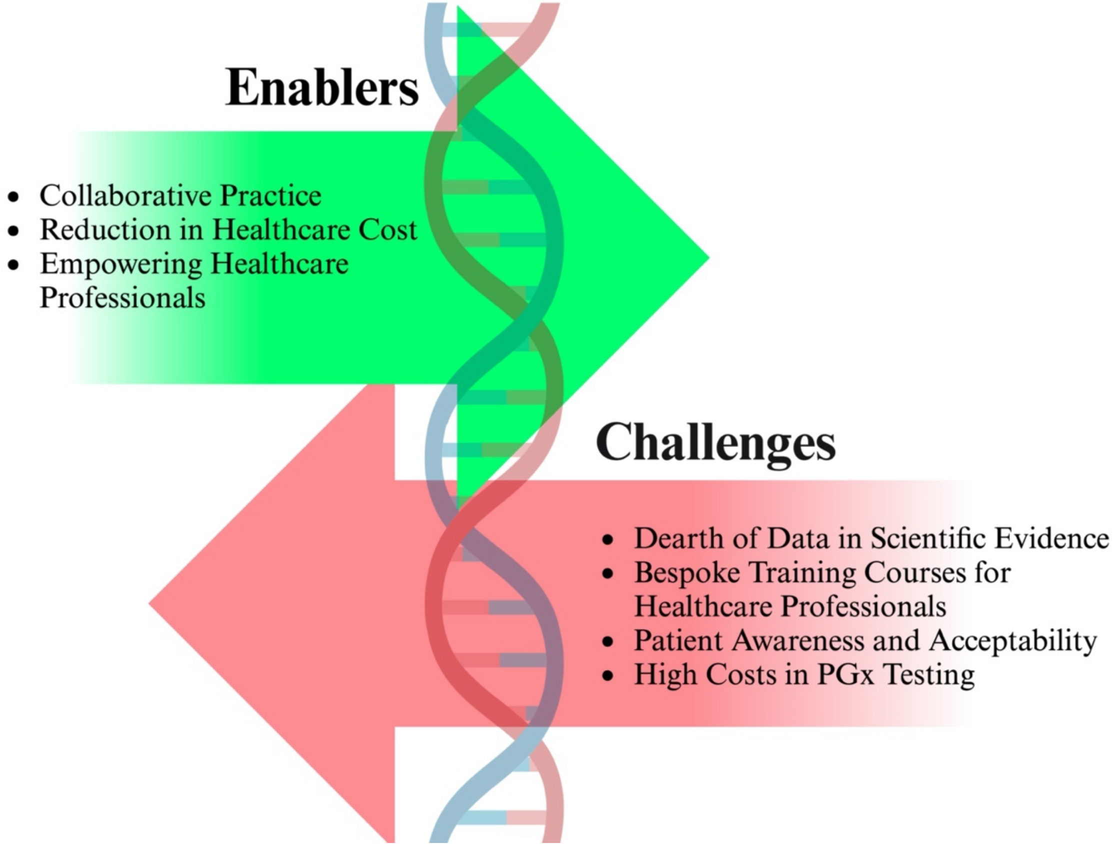

Supplement: online supplemental file 1 [file bmjopen-14-11-s001.pdf]
