# Supplementary material for: Scoping review of enablers and challenges of implementing pharmacogenomics testing in the primary care settings
Source: BMJ Open. 2024 Nov 5;14(11):e087064. doi: 10.1136/bmjopen-2024-087064 (PMC11552560; doi:10.1136/bmjopen-2024-087064)
Supplement: Supplementary file 2 [file bmjopen-2024-087064.reviewer_comments.pdf]

PEER REVIEW HISTORY

BMJ Open publishes all reviews undertaken for accepted manuscripts. Reviewers are asked to complete a checklist review form and are provided with free text boxes to elaborate on their assessment. These free text comments are reproduced below.

ARTICLE DETAILS

Title (Provisional)

A Scoping Review of Enablers and Challenges of Implementing Pharmacogenomics Testing in the Primary Care Settings

Authors

Mai, Chun-Wai; Sridhar, Sathvik B; Karattuthodi, Mohammed Salim; Ganesan, Perishithaa M; shareef, javedh; Lee, E Lyn; Armani, Keivan

---

VERSION 1 - REVIEW

**Reviewer**	**1**
**Name**	**Swen, Jesse**
**Affiliation**	**Leids Universitair Medisch Centrum**
**Date**	**22-May-2024**
**COI**	**none**

This scoping review of enablers and challenges of implementing PGx testing in primary care represents the result of sound scientific work and the authors should be applauded for their efforts. My main concern is that the current review has little to add to the scientific literature that is already available. The authors may consider the following points to improve their manuscript:

Minor comments:

** table 2 contains information on the included studies. However, key findings / conclusions for the individual papers are missing. Consider adding more details to this table

** please add the search string / key words that were used to identify included articles

** overall the paper would benefit from the addition of more specifics / details. The current descriptions are sometimes somewhat general

---

**Reviewer**	**2**
**Name**	**Cernat, Alexandra**

Affiliation McMaster University, Family Medicine

Date 06-Jul-2024

COI None.

There has been a great deal of literature examining barriers and facilitators of PGx testing implementation in the last few years; this review serves to bring this body of work together nicely and provide an overview of the knowledge in this area, specifically with respect to the primary care setting, thus far. This scoping review was conducted according to the framework by Arksey and O'Malley, as well as with guidance from the Joanna Briggs Institute and Levac and colleagues. I appreciate the authors' methodological rigour, but I do have questions and comments that I believe will strengthen this paper.

Background

- With respect to the penultimate sentence in the first paragraph of this section, can you elaborate on the use of the technologies you describe. Specifically, when you say that they have been "increasingly utilized" I was left wondering, utilized where and for what purpose? For example, in the clinical setting? In the context of research? This is a minor point and the addition of just a couple of words would be sufficient.

- While PGx testing can provide some guidance as to what medication(s) might be most appropriate for a particular patient, it is not a "silver bullet" and use of this testing does not necessarily mean that medical treatment will then be tailored to a person's unique genomic makeup. The phrasing of the last sentence in the first paragraph oversells this technology and I would recommend rephrasing and adding some more information to give a more accurate description what PGx testing can and cannot do.

- In the second paragraph, can you give a couple examples of types of frequently prescribed medications (eg., antidepressants, antibiotics) where genetic differences are known to play a role in inter-individual variances in drug response?

- It is certainly true that different PGx variants are more common in some populations than others, and this is incredibly important to consider when thinking about the implementation of this technology. However, you mention this idea once in the second paragraph of this section ("genetic differences within and between ethnic groups") and do not return to it again. Please make sure you either expand upon this in the background section or include a discussion about this in the discussion section. I would also encourage you to refrain from referring to "ethnic groups" – the issue here is not one of race or ethnicity, but rather of geography. I would refer instead to "geographical ancestry".

- What do you mean by "rational medicine utilization" in the third paragraph in this section (pg 3 line 48)?

- The sentence in the third paragraph that begins, "Not all insurance firms…." (pg 3 lines 53-54) is hard to parse. Can you please rephrase?

- When you say “active PGx testing” (pg 3 line 57), do you mean reactive testing?

- I would edit the last sentence on page 3 to reflect that both pre-emptive and reactive testing have been found to be cost-effective in different disease states or clinical care contexts.

- In the last paragraph on page 3, you bring in the idea that PGx testing has been adopted differently in different healthcare settings. Can you please expand on this? Because you focus specifically on primary care, it’s important that the reader understands why you have chosen to focus on this specific clinical care setting and what PGx testing in primary care currently looks like.

- Please restructure the second paragraph on page 4 (lines 16-36). You mention ethical concerns, then jump to barriers, then jump back to ethical concerns. This is hard to follow.

- In the second paragraph on page 4, you state that it is important to acknowledge ethical concerns, but you then only describe ethical concerns relevant to genetics and genomics in general. I would suggest that you focus your discussion of ethical issues on those concerns that are specific to or exacerbated by PGx testing.

- Can you expand on the idea of errors or misunderstandings in the third paragraph on page 4, and why this is particularly relevant to primary care?

Methods

- What specifically did you use from the Joanna Briggs Institute (page 4 line 56)?

- The information presented in the Selecting the Studies section (page 6) is typically presented in the first paragraph of the results section. I would suggest moving it there, and leaving just a description of your screening process in the methods.

- How did you identify the ten stakeholders that you consulted with (page 7)? It is important to consider not only how you determined the group of people who would be eligible to be a stakeholder in this part of the process, but also how you decided who to approach from within that group.

Results

- Can you please provide more detail as to the type of studies included (page 7)? An extremely wide array of study designs can fall under “quantitative studies” and “mixed methods studies”!

- The paragraph about stakeholders (page 8 lines 3-15) is unclear. What do you mean by “stakeholders”? Are these the participants included in the included studies?

- The last sentence in the first paragraph on page 8 is confusing and out of place.

- How did you define favourable/unfavourable/neutral (page 8 lines 20-23)? Especially since you later report that nearly all studies discussed the benefits of PGx testing in primary care?

- Can you please provide some commentary on the one study that does not mention benefits of PGx testing (page 8 line 49)?

- Can you expand on how cost-saving came up in the included studies (page 9 lines 22-32)? For example, did stakeholders discuss potential cost-savings as a perceived benefit of this technology, were economic evaluations included in this review, etc?

- More detail is needed in the paragraph about empowering healthcare professional to deliver their clinical services. For example, what are some of the identified benefits of implementation via community pharmacists (page 9 lines 40-42)?

- Cancer risk assessment is unrelated to PGx testing (page 9 line 47). Please remove that specific finding as it is separate from PGx testing.

- Please make sure your discussion of potential biases considers the study design of the papers in question (page 10 lines 10-20). For example, generalizability is not the aim of qualitative work, so qualitative papers should not be included in the n=18 that you report.

- Can you be more specific with respect to training for whom (page 10 lines 24-31)? Which types of healthcare providers (eg., primary care physicians, nurse practitioners, pharmacists, etc) specifically require more education on this topic?

Discussion

- Central to the discussion about privacy and implementation is the fact that in some jurisdictions, PGx testing is largely available only through commercial companies. This should also be touched upon in the discussion.

- Can you please cite some literature to support your discussion of patient concerns in the second paragraph on page 12?

Thank you for the opportunity to review this work.

VERSION 1 - AUTHOR RESPONSE

Dear Drs Partridge and Branch-Hollis,

Thank you for your consideration of the manuscript entitled “A Scoping Review of Enablers and Challenges of Implementing Pharmacogenomics Testing in the Primary Care Settings - bmjopen-2024-087064”. We highly value the time and effort spent by your office and your reviewers to propose suggestions to improve the above-mentioned manuscript. We present an itemised tabulated responses to the comments as per your request (in the attached file below) along with the track changed manuscript. We genuinely believe that by addressing the comments the manuscript has significantly improved.

We look forward to hearing from you. Please do not hesitate to contact us for any further queries.

**Reviewer 1 Comments**	**Our Response**
This scoping review of enablers and challenges of implementing PGx testing in primary care represents the result of sound scientific work and the authors should be applauded for their efforts. My main concern is that the current review has little to add to the scientific literature that is already available.	Thank you for your comments. As highlighted in the Background, PGx guided therapy is perceived to be more popular among the developed countries with variety acceptance among Europe countries. However, there is no comprehensive review to conclude whether such phenomena are scientifically evidenced in term of number of literatures. Our study, as highlighted in Figure 2, clearly highlighted the disparity of PGx among the developed countries. Also, studies related to PGx are mostly on enablers and challenges in tertiary hospitals, with limited comprehensive analyses targeting PGx in primary care. As far as we are aware, this is the only unbiased review that comprehensive look into the enablers and challenges of implementing PGx in primary care in order to add value to future policy decision and strategies to support PGx in primary care. Overall, the edits expand the results to highlight the novelty and impact of this study.
The authors may consider the following points to improve their manuscript:<br>Minor comments:<br>(1) table 2 contains information on the included studies. However, key findings / conclusions for the individual papers are missing. Consider adding more details to this table	Thank you for your comments. We agree that such details would be helpful to the readers. We have included the key findings in the revised Table 2. Editor has requested table 2 to be put under Supplementary File 2 since it is more than 2 pages.
(2) please add the search string / key words that were used to identify included articles.	Thank you for your comments. We have been added a supplementary file titled “Search Strategy”, listed as Supplementary File 1.
(3) overall the paper would benefit from the addition of more specifics / details. The current descriptions are sometimes somewhat general	Thank you for your comments. We agree that such details would be helpful to the readers. We have expanded our result sections and tables with more specific findings. Overall, the edits following addressing both reviewers’ comments have, hopefully, provided enough specifications and details.
**Reviewer 2 Comments**	**Our Response**
There has been a great deal of literature examining barriers and facilitators of PGx testing implementation in the last few years; this review serves to bring this body of work together nicely and provide an overview of the knowledge in this area, specifically with respect to the primary care setting, thus far. This scoping review was conducted according to the framework by Arksey and O’Malley, as well as with	Thank you for your kind and well-thought comments. We have amended all the concerns and we hope to receive your favorable reply on the amendment.

guidance from the Joanna Briggs Institute and Levac and colleagues. I appreciate the authors' methodological rigour, but I do have questions and comments that I believe will strengthen this paper.	
**Background**<br><br>- With respect to the penultimate sentence in the first paragraph of this section, can you elaborate on the use of the technologies you describe. Specifically, when you say that they have been "increasingly utilized" I was left wondering, utilized where and for what purpose? For example, in the clinical setting? In the context of research? This is a minor point and the addition of just a couple of words would be sufficient.	Thank you for your comments. We agree with your suggestion. We have amended the first paragraph to elaborate on the increasing use of health technologies in various PGx related discipline, namely the design of mutation-specific therapies, personalising early detection of disease strategies, personalising disease prevention, and personalising treatment have been increasingly utilised in both clinical settings and research contexts based on individual patient profiles. We also elaborated the paragraph with more details and discussion points to support the discussion:<br><br>"Pharmacogenomics (PGx) broadly defines how genomic variation affects a patient's response to a drug [1]. Distinct polymorphisms in drug-metabolising enzymes and drug transporters were a foundation for PGx[2]. With the advance in health technology, the 2000 collaborative effort to draft the human genome marked a turning point, followed by the International Single Nucleotide Polymorphisms (SNP) Map Working Group's efforts to map variations in the human genome sequence [2,3]. More importantly, the advancement of health technology has positioned PGx as a key component in the field of personalised medicine. The application of health technology has ranged from rationalising mutation-specific therapies to personalising early detection strategies, disease prevention, and treatments, have been increasingly utilised in both clinical settings and research contexts based on individual patient profiles [4]. This approach tailors medical treatment to an individual's unique genomic makeup to improve treatment outcomes and minimise adverse effects [5]."
- While PGx testing can provide some guidance as to what medication(s) might be most appropriate for a particular patient, it is not a "silver bullet" and use of this testing does not necessarily mean that medical treatment will then be tailored to a person's unique genomic makeup. The phrasing of the last sentence in the first paragraph oversells this technology and I would recommend rephrasing and adding some more information to give a more accurate description what PGx testing can and cannot do.	Thank you for your comments. We agree with your suggestion. The following paragraph has been revised: ". While PGx testing provides useful information by detecting genetic variants that impact medication metabolism and response, it is not ideal for all patients 6. PGx testing can help guide the selection of drugs that are more likely to be beneficial and have fewer adverse effects depending on an individual's genetic makeup 7. However, it does not consider other important aspects, such as the influence of environment, comorbid diseases, and patient adherence, which can substantially impact treatment results. As a result, while PGx testing is an effective tool for customizing therapy, it should be used with extensive clinical judgment rather than as a sole predictor of optimal treatment 8."
- In the second paragraph, can you give a couple examples of types of frequently prescribed medications (eg., antidepressants, antibiotics) where genetic differences are known to play a role in inter-individual variances in drug response?	Thank you for your comments. We agree with your suggestion. The following paragraph has been revised: "Genetic differences in drug-metabolising enzymes, transporters, receptors, and other therapeutic targets have been related to interindividual variances in the efficacy and safety of several frequently prescribed medications such as antidepressants (e.g., selective serotonin uptake inhibitors, SSRI) and anticoagulants (e.g., warfarin), which account for approximately 20-30% of medication

	response variability.”
- It is certainly true that different PGx variants are more common in some populations than others, and this is incredibly important to consider when thinking about the implementation of this technology. However, you mention this idea once in the second paragraph of this section (“genetic differences within and between ethnic groups”) and do not return to it again. Please make sure you either expand upon this in the background section or include a discussion about this in the discussion section. I would also encourage you to refrain from referring to “ethnic groups” – the issue here is not one of race or ethnicity, but rather of geography. I would refer instead to “geographical ancestry”.	Thank you for your comments. We agree with your suggestion. The following paragraph has been revised: “. Genetic differences do not follow a consistent pattern among populations. Instead, they show significant variation within and between different geographical ancestries 10. For example, specific PGx variants that impact drug metabolism are more commonly found in certain populations, leading to variations in drug response and the occurrence of adverse effects. Acknowledging and understanding these genetic variations specific to different populations is essential for the successful application of personalised medicine. This knowledge enables clinicians to customize treatments that are safe and effective for a wide range of patients 11,12. Inter-individual genetic differences within and between geographical ancestry contribute significantly to medication response variability and are linked to variants affecting the pharmacokinetics (PK) and pharmacodynamics (PD) of drugs 13,14.”
- What do you mean by “rational medicine utilization” in the third paragraph in this section (pg 3 line 48)?	Thank you for your comments. We agree with your suggestion. The term rational medicine utilization is changed to “promotion of appropriate and evidence-based medication usage
- The sentence in the third paragraph that begins, “Not all insurance firms….” (pg 3 lines 53-54) is hard to parse. Can you please rephrase?	Thank you for your comments. We agree with your suggestion. The sentence is rephrased for more clarity to: “Few insurance firms offer coverage for PGx testing, and those that do must follow strict guidance and policies to justify and approve requested PGx tests.”
- When you say “active PGx testing” (pg 3 line 57), do you mean reactive testing?	Thank you for your comments. Yes, we meant reactive testing! This has been corrected.
- I would edit the last sentence on page 3 to reflect that both pre-emptive and reactive testing have been found to be cost-effective in different disease states or clinical care contexts.	Thank you for your comments. We agree with your suggestion. The following paragraph has been revised: “Both pre-emptive and reactive testing have been found to be cost-effective in different disease states or clinical care contexts and positively impact patient outcomes.”
- In the last paragraph on page 3, you bring in the idea that PGx testing has been adopted differently in different healthcare settings. Can you please expand on this? Because you focus specifically on primary care, it’s important that the reader understands why you have chosen to focus on this specific clinical care setting and what PGx testing in primary care currently looks like.	Thank you for your comments. We agree with your suggestion. We have added an observational study stating the importance of PGx testing in the primary care setting. The following paragraph has been revised:<br><br>“An observational study from the United Kingdom discussed the implementation of PGx testing in secondary care for high-risk medications. The authors emphasised the need for broader application in primary care owing to the high prescribing tendency in the community [26] . The adoption of PGx

	testing services in different healthcare settings has varied owing to a multitude of factors, including the promotion of appropriate and evidence-based medication usage, ethical considerations, legal implications, healthcare provider and patient education, support for electronic health records, clinical utility and validity of test outcomes, accessibility, regulatory frameworks, as well as availability and affordability.
- Please restructure the second paragraph on page 4 (lines 16-36). You mention ethical concerns, then jump to barriers, then jump back to ethical concerns. This is hard to follow.	Thank you for your comments. We agree with your suggestion. The paragraph was restructured as below:<br><br>"An observational study from the United Kingdom discussed the implementation of PGx testing in secondary care for high-risk medications. The authors emphasised the need for broader application in primary care owing to the high prescribing tendency in the community [26] . The adoption of PGx testing services in different healthcare settings has varied owing to a multitude of factors, including the promotion of appropriate and evidence-based medication usage, ethical considerations, legal implications, healthcare provider and patient education, support for electronic health records, clinical utility and validity of test outcomes, accessibility, regulatory frameworks, as well as availability and affordability. [20,27–30]. The cost implications of PGx testing depend on the insurance coverage offered by companies. Few insurance firms offer coverage for PGx testing, and those that do must follow strict guidance and policies to justify and approve requested PGx tests [31]. This can affect the preference for pre-emptive PGx and reactive PGx testing [32]. Both pre-emptive and reactive testing have been found to be cost-effective in different disease states or clinical care contexts and positively impact patient outcomes [33]."
- In the second paragraph on page 4, you state that it is important to acknowledge ethical concerns, but you then only describe ethical concerns relevant to genetics and genomics in general. I would suggest that you focus your discussion of ethical issues on those concerns that are specific to or exacerbated by PGx testing.	Thank you for your comments. We agree with your suggestion. The paragraph was restructured as below:<br><br>"While PGx testing offers several benefits, it is important to acknowledge the ethical concerns surrounding it, especially in a primary care setting. Ethical dilemmas may emerge due to the potential misuse of informed consent in genomic testing, including the potential dangers, risk, harms, and consequences associated with genomic information [37,38]. Additionally, genomic information may raise questions about ownership, access rights, affordability, fiduciary responsibility, respect, and the possibility of discrimination [37,38]. Furthermore, there are concerns about the administering PGx testing among vulnerable communities. Assessing the potential long-term implication of identifying genomic variability in different categories of vulnerable population may raise ethical concerns. [37–39]."
- Can you expand on the idea of errors or misunderstandings in the third paragraph on page 4, and why this is particularly relevant to primary care?	Thank you for your comments. We agree with your suggestion. The paragraph has been modified as below:<br><br>"Moreover, since the interpretation of genomic information is still evolving, inadequate inferences or confounding factors may cause healthcare providers to opt for incorrect treatment, complicating

	the ethical landscape and raising public concern about their health [43]. While PGx testing offers positive benefits, it is important to acknowledge the concerns related to this practice, especially in a primary care setting. Thus, this scoping review was conducted to systematically review the current state of PGx in the primary care and determine the enablers as well as challenges of implementing PGx testing in primary care settings.
**Methods**<br><br>- What specifically did you use from the Joanna Briggs Institute (page 4 line 56)?	Thank you for your comments. We used the Joanna Briggs Institute 6-steps in this study. They are:<br>1- Identifying the review question<br>2- Identifying the relevant studies<br>3- Selecting the studies<br>4- Charting the data<br>5- Collating, summarising, and reporting the results<br>6- Consulting stakeholders<br>Numbers are added to each of the 6 steps for more clarity.
- The information presented in the Selecting the Studies section (page 6) is typically presented in the first paragraph of the results section. I would suggest moving it there and leaving just a description of your screening process in the methods.	Thank you for your comments. We agree with your suggestion. The information have been moved to the beginning of the results section, whilst a methodological description is left in the methods section. The following paragraph has been revised: “Articles were identified across five databases, which were exported into Covidence™ for further processing. Two reviewers independently screened each article, and a third reviewer resolved any discrepancies.”
- How did you identify the ten stakeholders that you consulted with (page 7)? It is important to consider not only how you determined the group of people who would be eligible to be a stakeholder in this part of the process, but also how you decided who to approach from within that group.	Thank you for your comments. We agree with your suggestion. The following paragraph has been revised:<br>“Although stakeholders' involvement and consultation are not mandatory stages for conducting scoping reviews, we involved a subset of stakeholders who were available to us in two stages. These stakeholders were the primary care physicians or community pharmacists who were elected leaders in their respective professional societies and had at least 10 years primary care clinical experience. Invitations were sent by the research team to all eligible stakeholders. All stakeholders who declared no conflict of interests with any PGx service provider were to participate. We conducted a brainstorming session with these stakeholders. The ten stakeholders were from independent or chain medical clinics (n = 5) or community pharmacies (n = 5). We then presented the findings to them for their comments and feedback.
**Results**	Thank you for your comments. We agree with your suggestion. The following information were

	included :
- Can you please provide more detail as to the type of studies included (page 7)? An extremely wide array of study designs can fall under "quantitative studies" and "mixed methods studies"!	"The study types can be categorised into Quantitative Studies (n = 16) and Mixed Method Studies (n = 54). Quantitative studies can be further divided into (i) Randomized Controlled Trials (n = 5), wher the controlled experimental settings were used to assess the efficacy of PGx testing; (ii) Cohort Studies (n = 4), where these groups were monitored over time to evaluate the outcomes of PGx testing; (iii) Cross-Sectional Surveys (n = 3), where one-time data collection methods were used to evaluate respondents' beliefs, expertise, and PGx-related behaviour; (iv) Case-Control Studies (n = 2) where the effects of PGx testing were examined by comparing individuals with particular results to those without; and (v) Pre-Post Intervention Studies (n = 2), where the outcomes were examined both before and after PGx testing was used.<br><br>In addition, Mixed Methods Studies (n = 54) can be further categorised into (i) Explanatory Sequential Designs (n = 15), where quantitative data were gathered first, followed by qualitative data to explain the quantitative results; (ii) Exploratory Sequential Designs (n = 20), where quantitative data were collected after conducting qualitative research to create or refine hypotheses; and (iii) Convergent Parallel Designs (n = 19),where qualitative and quantitative data were gathered concurrently, the finding were compared and comprehensive conclusions were drawn. This thorough analysis addresses the variability within the broader categories of quantitative and mixed methods research, providing a deeper understanding of the studies covered in the study."
- The paragraph about stakeholders (page 8 lines 3-15) is unclear. What do you mean by "stakeholders"? Are these the participants included in the included studies?	Thank you for your comments. Stakeholders (referred in the original text's page 8, lines 3) are the respective public or professionals cited in the literature. The paragraph has been revised to ensure clarity :<br><br>"From the selected literature, the stakeholders included the service users/patients, members of the public, healthcare professionals including general practitioners, physicians, pharmacists, nurses, physician assistants, public health consultants/professionals, geneticists, phlebotomists, genetic counsellors, mental health providers, obstetricians, gynaecologist, psychiatrists, and cardiologist. Most of the stakeholders were primary care physicians (n = 43), followed by pharmacists (n = 32), allied healthcare professionals (n = 27), and primary care providers who were not specified (n = 15). Additionally, there was general agreement with the results when they were presented to a panel of stakeholders (n = 10) whom we had individually consulted for this study."
- The last sentence in the first paragraph on page 8 is confusing and out of place.	Thank you for your comments. We agree with your suggestion. The following paragraph has been revised:<br><br>"Additionally, there was general agreement with the results when they were presented to a panel of stakeholders (n = 10) whom we had individually consulted for this study."

- How did you define favourable/unfavourable/neutral (page 8 lines 20-23)? Especially since you later report that nearly all studies discussed the benefits of PGx testing in primary care?	Thank you for your comments. We apology for missing out this information. We have added the definition for favourable, unfavourable, and neutral views in page 8. The following paragraph has been revised :<br><br>"To understand the current status of PGx testing in primary care, we classified the key conclusion of these studies into 3 categories, namely the (i) favourable view in which the key conclusion supports PGx implementation in primary care; (ii) not favourable, in which the key conclusion does not support PGx implementation in primary care; and (iii) neutral views in which the study did not provide a clear stance on supporting or not supporting PGx implementation in primary care."
- Can you please provide some commentary on the one study that does not mention benefits of PGx testing (page 8 line 49)?	Thank you for your comments. We agree with your suggestion. We had expanded the description of the study in the revised text. The following paragraph has been revised:<br><br>"Specifically, Türkmen D et al highlighted PGx results could be guided by databases such as PharmGKB, which contains studies with low or moderate level of evidence. They also noted that the study design, with qualitative studies not being meantfor generalisability of the findings, along with factors such as diverse ethnicity, heterogeneity, poor compliance to medication, statistical bias and publication [48]."
- Can you expand on how cost-saving came up in the included studies (page 9 lines 22-32)? For example, did stakeholders discuss potential cost-savings as a perceived benefit of this technology, were economic evaluations included in this review, etc?	Thank you for your comments and insightful feedback. We agree with your suggestion. To clarify the discussion on cost-saving aspects related to pharmacogenomics (PGx) testing in the review, I have expanded on how cost-saving features were addressed in the included studies. The revised section is as follows:<br><br>"The potential for cost-saving associated with the implementation of PGx testing was mentioned in 20% of the studies (n = 15). Various cost-saving approaches were proposed, namely (i) economic evaluations; (ii) stakeholders perceptions; and (ii) indirect evidence. Formal economic evaluations were used in several studies to determine whether PGx testing was cost-effective. Cost-utility, cost-benefit, and cost-effectiveness studies were frequently performed as part of these assessments. For example, when PGx testing guided drug selection and dose decisions, a randomized controlled trial found lower healthcare expenditures due to fewer adverse drug events (ADEs) occurred. This study measured the financial gains connected with fewer ADEs and hospitalizations using a cost-effectiveness methodology [56]. An economic evaluation approach was employed in prospective cohort research conducted in Singapore to evaluate the effects of a PGx-based medical decision support system on healthcare expenditures and quality. The study showed that by enhancing medication dosage and improving treatment results, PGx testing led to cost savings [57].<br><br>In term of stakeholder perceptions, some research examined cost-savings from the viewpoint of

	stakeholders, such as legislators and healthcare professionals, in addition to economic evaluations. Stakeholders believed that PGx testing could be an effective way to reduce overall healthcare costs by minimizing trial-and-error prescribing and the adverse drug experiences that come with it. Qualitative interviews with primary care physicians, for instance, revealed that PGx testing could save long-term expenses by enabling more accurate medication administration. Alternative approaches would be through indirect evidence. A few studies highlighted improvements in patient outcomes that were associated with lower healthcare utilization, which served as an indirect source of cost-saving data. These studies suggested that more targeted treatments resulting from PGx testing could reduce total healthcare costs by avoiding the need for extra interventions, even though they did not conduct direct economic evaluations.
- More detail is needed in the paragraph about empowering healthcare professional to deliver their clinical services. For example, what are some of the identified benefits of implementation via community pharmacists (page 9 lines 40-42)?	Thank you for your valuable feedback. We agree with your suggestion. To address the need for more detail regarding the benefits of empowering healthcare professionals, particularly community pharmacists, to deliver clinical services through pharmacogenomics (PGx) testing, we have expanded the relevant sections. The following paragraph has been revised :<br><br>"Over 28% studies emphasized the importance of incorporating healthcare professionals such as community pharmacists, to improve patient care through implementing PGx in a primary care setting. The advantages of involving community pharmacists in administering PGx testing include (i) enhanced medical management, (ii) increased accessibility and patient engagement, (iii) better integration with clinical decision support systems, and (iv) increased physician adoption of PGx. By using PGx testing, community pharmacists can customize more drug regimens based on each patient's unique genetic profile, leading to fewer adverse drug reactions (ADRs) and increased efficacy. An open-label, non-randomized observational trial reported better patient outcomes from community pharmacists based PGx screening, since pharmacists could efficiently provide more input on the regimens [58].<br><br>In addition, patients' accessibility to PGx testing is increased when it is incorporated into community pharmacy practices, especially in underprivileged areas. Research indicated that patients whom experienced easier access to genomic services via their neighbourhood pharmacies, were likely to have thoughtful and educated conversations regarding their treatment options [59] . Community pharmacists play a crucial role in helping patients understand the meaning of PGx test results. Patients would then adhere to the individualized treatment programs when they are more educated about how genetic information can guide their pharmaceutical choices.<br><br>Interesting, including PGx testing in a clinical decision support system (CDSS), greately enhanced its efficacy. Research indicated that community pharmacists who used CDSSs in combination with PGx testing were more capable of making well-informed choices regarding medication dosage and therapy modifications. This integration reduces the possibility of drug errors and helps provide more

	accurate recommendations [59] . Moreover, physician preference for PGx in patient care has increased due to the convenience of receiving PGx services through community pharmacists. By collaborating with pharmacists, who perform PGx testing, physician can focus on better decision-making, and ultimately improves patient outcomes [60]."
- Cancer risk assessment is unrelated to PGx testing (page 9 line 47). Please remove that specific finding as it is separate from PGx testing.	Thank you for your comments. We agree with your suggestion. The sentence has been removed.
- Please make sure your discussion of potential biases considers the study design of the papers in question (page 10 lines 10-20). For example, generalizability is not the aim of qualitative work, so qualitative papers should not be included in the n=18 that you report.	Thank you for your comments. We agree with your suggestion. The following paragraph has been revised:<br>"Almost a quarter (n = 18) of the studies also acknowledged that their studies may had the inevitable recruitment bias, which could limit the potential to immediately implement PGx findings across all populations in primary care settings."
- Can you be more specific with respect to training for whom (page 10 lines 24-31)? Which types of healthcare providers (eg., primary care physicians, nurse practitioners, pharmacists, etc) specifically require more education on this topic?	Thank you for your comments. We agree with your suggestion. We have included more precise information about the requirement for customized PGx training modules for various categories of healthcare professionals. The following paragraph has been revised:<br>"The insufficiency of appropriate training for primary care providers to administer PGx testing was a notable obstacle identified in 17 studies. Each healthcare practitioner have distinct PGx training. Due to their limited exposure to genetic concepts and how they are applied in daily practice, many primary care physicians (PCPs) report feeling unprepared to use PGx testing. PCPs need comprehensive primary care training to evaluate PGx test results and incorporate them into clinical decision-making. Training courses must concentrate on managing drug-gene interactions, using genetic information to inform medication selection and dosage, and clearly communicating findings to patients. Nurse practitioners' capacity to offer effective patient education and individualized medication management is hampered by their lack of PGx testing-specific training such as data analysis, and the incorporation of PGx data into patient care plans. In addition, the limited availability of specialised training programs for pharmacists also hinders their ability to apply PGx testing in their practice [62]. Specific trainings for pharmacists should include interpreting of genetic data, applying PGx in drug therapy management, and integrating into pharmacy practice. The inadequacy of customized training programs for these diverse healthcare worker groups limits their ability to apply PGx testing in primary care environments. Addressing this gap with focused educational initiatives is essential to optimizing the benefits of PGx technology.
**Discussion**	Thank you for your comments. Yes, it is a great concern. We have incorporated the statement and have recommended for reviewing and updating the existing Data Privacy Act and rules for improving

- Central to the discussion about privacy and implementation is the fact that in some jurisdictions, PGx testing is largely available only through commercial companies. This should also be touched upon in the discussion.	the public preferences toward PGx testing. The following paragraph has been revised:<br>"Commercial companies' access to patients' genetic data is also a concern, hence the need for reviewing and updating the existing data privacy act and rules to improve the public preferences towards PGx testing [65]. Building trust and addressing these concerns is essential for promoting patient acceptability of PGx testing. Tailoring educational materials and communication strategies to meet the needs of diverse patient populations is crucial for promoting awareness and acceptability of PGx testing."
- Can you please cite some literature to support your discussion of patient concerns in the second paragraph on page 12?	Thank you for your comments. We have added the following three references regarding the "privacy and genetic information", namely:<br>82. Stiles D, Appelbaum PS. Cases in Precision Medicine: Concerns About Privacy and Discrimination After Genomic Sequencing. *Ann Intern Med*. 2019;170(10):717-721. doi:10.7326/M18-2666<br>83. Clayton EW, Halverson CM, Sathe NA, Malin BA. A systematic literature review of individuals' perspectives on privacy and genetic information in the United States. *PloS One*. 2018;13(10):e0204417. doi:10.1371/journal.pone.0204417<br>84. Clayton EW, Evans BJ, Hazel JW, Rothstein MA. The law of genetic privacy: applications, implications, and limitations. *J Law Biosci*. 2019;6(1):1-36. doi:10.1093/jlb/lsz007

VERSION 2 - REVIEW

**Reviewer**	**2**
**Name**	**Cernat, Alexandra**
**Affiliation**	**McMaster University, Family Medicine**
**Date**	**24-Sep-2024**
**COI**	**I declare no competing interests.**

The authors have addressed all of my previous comments. I have no further suggestions.
